# Dayara bugyal restoration model in the alpine and subalpine region of the Central Himalaya: a step toward minimizing the impacts

**DOI:** 10.1038/s41598-021-95472-y

**Published:** 2021-08-16

**Authors:** Jagdish Chandra Kuniyal, Priyanka Maiti, Sandeep Kumar, Anand Kumar, Nisha Bisht, K. Chandra Sekar, Satish Chandra Arya, Sumit Rai, Mahesha Nand

**Affiliations:** 1 G. B. Pant National Institute of Himalayan Environment, Kosi-Katarmal, Almora, 263 643 Uttarakhand India; 2 Divisional Forest Office, Tarai East Forest Division, Uttarakhand Forest Department, Haldwani, Uttarakhand 263 139 India

**Keywords:** Restoration ecology, Conservation biology

## Abstract

Eco-restoration initiative work in the high altitude Dayara pastureland (3501 m) from the Indian Himalayan Region has been considered to be one of the successful field demonstration against both natural and anthropogenic degradation. The present study therefore attempts to assess the implications of entire eco-restoration model as practiced by Department of Forest, Government of Uttarakhand in 2019. Its assessment was done by calculating restoration success index by way of considering three categories, viz*.*, direct management measure (M), environmental desirability (E) and socio-economic feasibility (SE) considering 22 individual variables. ‘M’ comprised both biotic and abiotic pressures. Grazing and tourism were biotic, while abiotic pressure was considered mainly soil erosion in alpine area due to topographic fragility. Above ground vegetation profile and below ground soil nutrient profile (N, P, K, pH and water holding capacity) were analyzed in ‘E’ component. In the last but not least, ‘SE’ was analyzed to assess the social acceptability of the local communities and stakeholders who are supposed to be ultimate beneficiary of alike interventions. Direct management measure was found with the variable index score of 0.8 indicating the higher score as compared to environmental desirability (0.56). Under direct management measure, grazing and tourists’ carrying capacity of the area was analyzed with high management needs to call the region sustainable in terms of availability of bio-resources. The ecosystem index score was evaluated for the reference (81.94), treated (64.5) and untreated zones (52.03), wherein increasing profile of these values were found. The outcomes like improved vegetation profile in terms of total herb density, soil nutrient profile of the restored area along with soil pH (4.96) and water holding capacity (49.85%) were found to be restored significantly along with controlling 169.64 tonne year^-1^ soil erosion from draining. The assessment of grazing pattern of 118 migratory Cow Unit (CU) (76 horse/mule and 18 sheep/goat, already controlled), 318 local CU (30 horse/mule and 187 sheep/goat) were calculated and recommended to be controlled. Tourists’ carrying capacity of 274 tourists per day and manual removal of *Rumex nepalensis* at the shepherd camping site were found to be worth to apply in the area. Use of biodegradable but locally sourced material and engaging local villagers in this endeavor were also found to be in harmony with SDG Goal 1 (no poverty). Therefore, the restoration and its evaluation model could have its future prospects to prove as a successful restoration practice. This restoration practice could not only be worth in high altitude degraded alpine pastures of the Indian Himalayan Region but also to other mountain alpine and sub-alpine ecosystems.

## Introduction

In the present scenario, gradual degradation of high elevation mountain eco-systems and their rational ecological management have been a matter of great concern^1^. Although several restoration efforts have been made in high altitude forests, treeline zone and wetland, yet restoration in meadows especially in alpine and sub-alpine have not been much reported so far as they are unique, complex, and fragile natural area associated with restoration implementation difficulties^2–4^.

Land restoration in high altitude degraded pasture areas addresses diverse issues associated with Sustainable Development Goals (SDGs). As restoration activities produce employment, thus improving the socioeconomic conditions of the poor (SDG-1, no poverty) needs a priority. Its successful examples include use of bamboo for land restoration in China, Ethiopia, Cameroon, Vietnam, India, Madagascar, Ghana, the Philippines and Kenya^5^. Other co-benefits of land restoration include increased community resilience (SDG-2, zero hunger), improved good health and well-being (SDG-3) of the associated villages, impacts on access to quality education (SDG-4, quality education), income generation for women villagers (SDG-5, gender equality), restores ground water and in turn ensure future clean water availability (SDG-6, clean water and sanitation), and also have direct impact on SDG-13 (climate action), SDG-15 (life on land) and SDG 17 (partnerships to achieve the goal) (United Nations Environment Programme, 2020). In context to land restoration, degradation of high altitude pastureland leading to soil erosion nowadays have become a matter of great concern. Some of the examples of such lands include, montane grasslands of the north-eastern Italian Alps (Italy)^6^, Asteroussia Mountains (Greece)^7^, and High Himalayas in Nepal and India^8^.Therefore, restoration of alpine pastures is a global need. Some restoration guidelines available for low altitude forest and degraded lands include Restoration Opportunities Assessment Methodology (ROAM)^9^, International principles and standards for the practice of ecological restoration, Ecological Restoration Guidelines for British Columbia^10^ and Assessing Landscape Restoration Opportunities for Uttarakhand, India, etc.^11^.

Some examples of the restoration activities of Govind Ballabh Pant National Institute of Himalayan Environment (GBP-NIHE), Kosi, Almora, Uttarakhand, India have been implemented in Indian Himalayan Region include Sloping Watershed Environment Engineering Technology (SWEET) developed by GBP-NIHE in 1994, Badrivan Restoration Programme (BRP) at Badrinath, Uttarakhand in 1993, development of an agroforestry model at Bansbara village, Rudraprayag District Uttarakhand in 2001, forest eco-restoration programme at Kolidhaik, Lohaghat, Uttarakhand in 2004, community wasteland (open grazing land) restoration at Arah village in 1992, Bageshwar District, Uttarakhand, silvi-pasture development in Uttarakhand, rehabilitation of Bhimtal lake, Nainital, Restoration of Surya Kunj at Katarmal, Almora, Uttarakhand, Dhoranalla and Mohal *Khad* (seasonal stream) restoration work in Mohal, Kullu, Himachal Pradesh, implementation of Contour Hedgerow Farming System Technology (CHFST) in Sikkim in 2005, and rehabilitation of degraded community land in Gumod, Champawat district, Uttarakhand^12^.

Still restoration activities in high altitude degraded grasslands have neither proper guideline nor field examples available till date. In this regard, the present work is a field illustration of eco-restoration in high altitude grassland at Dayara alpine pasture (3501 m) which was initiated by the Department of Forest, Govt. of Uttarakhand. So, the present attempt aims at evaluation of its impact in terms of grazing capacity, carrying capacity of tourists, overall land stabilization from soil erosion, vegetation profile especially plant growth, soil nutrients availability, etc. The applied restoration approach for current work is in harmony with the “Scientific Conceptual Framework for Land Degradation Neutrality” by the United Nations Convention to Combat Desertification (UNCCD) Science-Policy Interface^13^, and also concerns restoration definitions established by the Society for Ecological Restoration^14^.

## Study area

**Figure 1:**
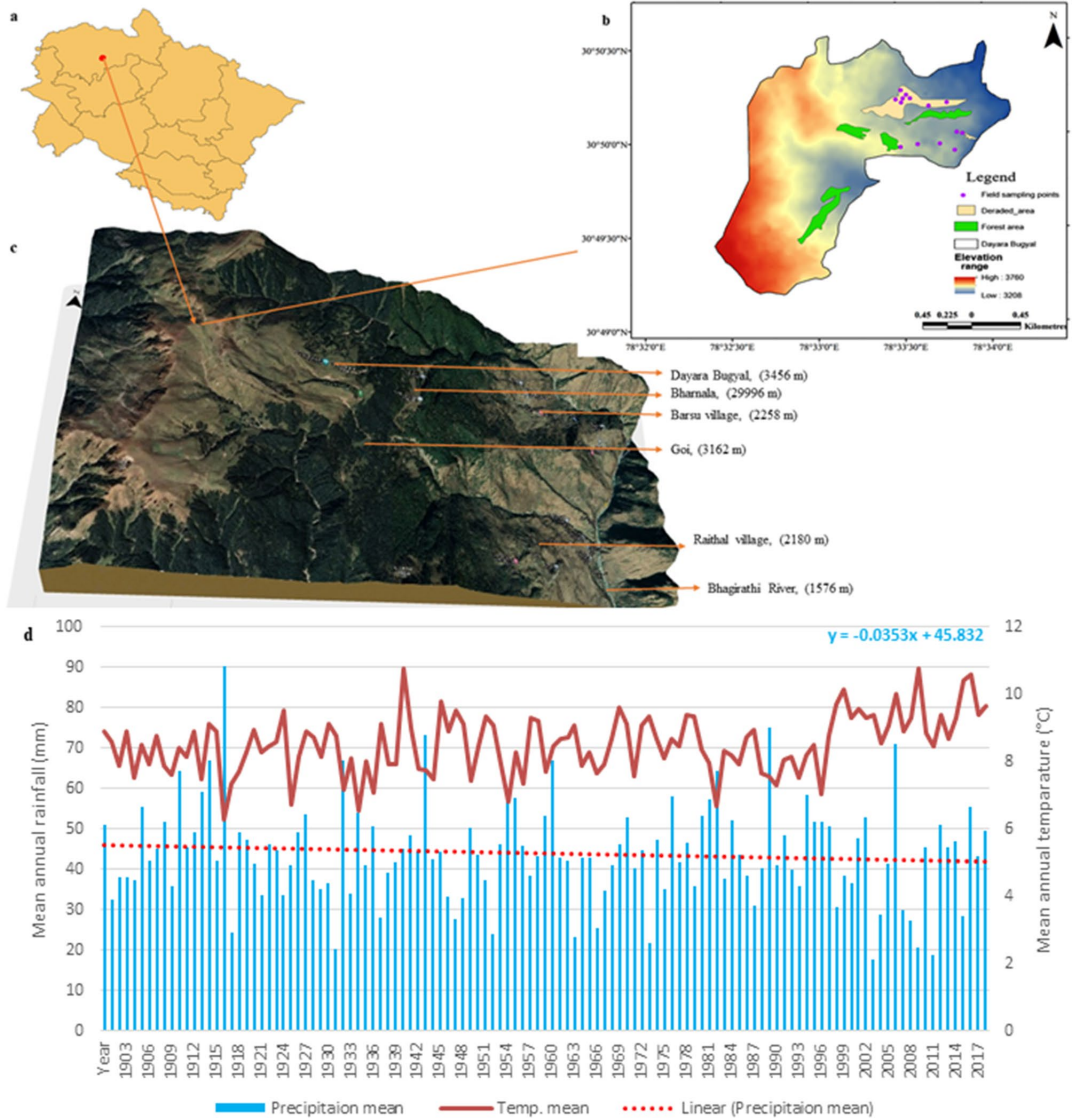
(**a**, **b**, **c**) Geographical extent, and (**d**) climatic profile of the study area. {Source: CRU TS 4.04 (land), 0.5°}.

The study area of the current work is the Dayara alpine meadow (3501 m) from Uttarkashi district of Uttarakhand, India. The Dayara bugyal lies from 30°49′18.53"N to 78°32′31.20"E to 30°50′31.82"N to 78°33′24.71"E and covers an area of 3.38 sq km (Fig. 1). The entire area comprises extraordinary ecological diversity with a large adjoining regions that helps in maintaining significant biodiversity of both the Himalayan wildlife flora and fauna. The pasture area is mainly dominated by three types of vegetation, namely, herbaceous meadow (*Rumex nepalensis* Spreng., *Anaphalis cuneifolia* (DC.) Hook. f., *Hackelia uncinata* (Royle *ex* Benth.) C.E.C. Fisch.*,*etc.), shrubberies (*Rhododendron anthopogon* D. Don—*Rhododendron campanulatum* D. Don) and stable boulder (*Bergenia strachyei* (Hook. f. & Thomson) Engl., *Arnebia benthamii* (Wall. *ex* G. Don) I.M. Johnst., etc.)^15^. Faunal diversity of the area consists of the Himalayan wildlife species like Musk dear (*Moschus leucogaster*, Hodgson, 1839), Brown bear (*Ursus arctos isabellinus* Horsfield, 1826), Himalayan Thar (*Hemitragus jemlahicus* C.H. Smith, 1826), Monal Pheasant (*Lophophorus impejanus* Latham, 1790), etc. It is the origin site of two important tributaries of River Bhagirathi, locally known as Papad Gad (local stream) and Swari Gad. Both the streams not only provide drinking and irrigation water to the downstream villages of Raithal, Kyark and Barsu but also maintain the geo-hydrological system of the entire area. The place is also famous for its natural beauty and a favourable tourist spot which have led to a variety of adverse impacts like increase in solid waste, trampling due to night camps and other anthropogenic activities. Apart from these, the ecosystem has experienced the adversities of climate change as heavy rainfall, flash floods, cloudbursts, etc. leading to disasters in the lower catchment of the Bhagirathi valley. Along with this, the nomadic tribe known as Gujjars seasonally migrate to this pastureland for grazing with the onset of spring and stay there till autumn. The unattended cattle of the adjacent villages also occupy the area in spring and summers resulting in habitat degradation^16^. Gradual increase in mean annual temperature and decrease in mean annual precipitation was also reflected from the climatic profile of the area with gridded resolution 0.5 x 0.5° (CRU TS 4.04, land) (Supplementary Table S1, S2).

## Results

### Direct management measure (M)

**Figure 2:**
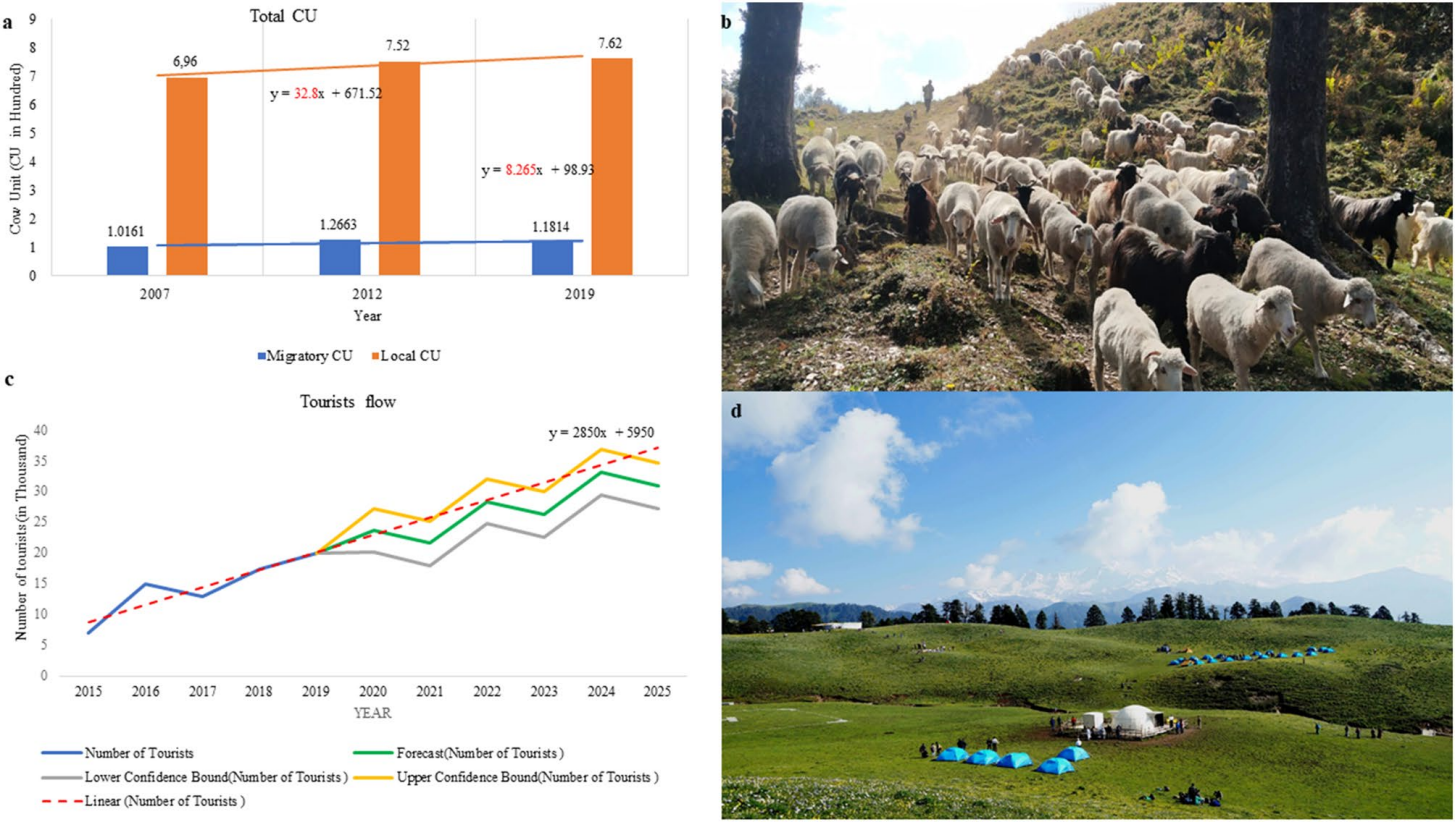
Anthropogenic interference at Dayara bugyal: (**a**) total cow unit of the area from 2007 to 2019, (**b**) return of animals from the Dayara bugyal before upcoming winter season, (**c**) time series analysis of tourists’ influx in the Dayara (2015–2025), and (**d**) tourists’ influx and their camping in the Dayara before the restoration period (2d-Photo credit: Mr.Santosh Saklani, Uttarakashi).

Grazing capacity of the Dayara bugyal was calculated by evaluating the standard forage production and daily demand of the livestock population. Total 70 species were found in the sampled grazing areas dominated by *Anaphalis cuneifolia*, *Taraxacum officinale* W.W. Weber ex F.H. Wigg., *Iris kemaonensis* Wall. *ex* Royle, *Sibbaldia parviflora* Willd., *Kobresia nepalensis* (Nees) Kk., *Trifolium repens* L., *Danthonia cachemyriana* Jaub. & Spach, *Carex nubigena* D. Don, etc. Among the studied vegetation, 37 dominant palatable species recorded with the 52% of the total number of studied species (palatable & unpalatable) were selected for calculation of forage production (Supplementary Table S3). Total dry forage yield of the Dayara bugyal was calculated 10,003 quintal per year (Table 1) and yield of standard dry forage was 5001 quintal per year which was 50% of the total yield for 4.23 sq km area. Only suitable area for grazing was considered for the present work. Intake of one Cow Unit (CU) was calculated 7.5 kg dry matter per day and 153 (May–September) days grazing time was taken into account. The grazing capacity of the area was calculated as 436 cow unit per year. Total migratory animal data for Taknor range and livestock census data for the village livestock were found increasing in last 10 years (Supplementary Table S4). As a part of management measure, grazing camps of Barsu villagers were then shifted away from Dayara to Lambidhar. Migratory animals were also found to be reduced reasonably in the Tanknor range. According to the migratory animal record of Forest Department Uttarkashi (2020) and livestock census data, total 881 CU were found to be grazed in the area in the year 2019. This value exceeds the grazing capacity but the numbers of migratory animals (Gujjars) were found to be continuously decreasing at a rate of 20 CU per year (Fig. 2).

**Table 1:** Standard forage production in the Dayara alpine pastureland.

Plant name	Density (individual/m^2^)*	Above ground biomass (g/m^2^)*	Forage yield (quintal /year)
*Aconogonum tortuosum* (D. Don) Hara	0.4	4.2	177.86
*Anemone obtusiloba* D.Don	0.23	2.68	113.49
*Arnebia benthamii* (Wall. *ex* G. Don) I.M. Johnst.	0.06	12.8	542.04
*Bistorta vivipara* (L.) Gray	9.8	16.8	711.43
*Bupleurum longicaule* Wall. *ex* DC	0.2	1.3	55.05
*Carex nubigena* D. Don	4.42	1.5	63.52
*Carex setigera* D. Don	0.82	7.2	304.90
*Cyananthus lobatus* Wall. *ex* Benth.	0.57	6.1	258.32
*Dactylorhiza hatagirea* (D.Don) Soo	0.23	1.3	55.05
*Danthonia cachemyriana* Jaub. & Spach	0.33	60.2	2549.30
*Epilobium latifolium* L.	0.24	1.1	46.58
*Eritrichium canum* (Benth.) Kitam.	0.62	1.2	50.82
*Euphorbia stracheyi* Boiss.	0.2	1.4	59.29
*Galium rotundifolium* L.	0.23	0.2	8.47
*Gentiana argentea* (D.Don) Griseb.	0.23	0.8	33.88
*Geranium wallichianum* D.Don *ex* Sweet	0.3	0.9	38.11
*Geum elatum* Wall. *ex* G. Don	0.2	1.2	50.82
*Impatiens scabrida* DC.	0.31	1.3	55.05
*Origanum vulgare* L.	1.22	4.3	182.09
*Oxygraphis polypetala* (D.Don) Hook.f. & Thomson	0.02	1.4	59.29
*Parnassia nubicola* Wall. *ex* Royle	0.27	3.4	143.98
*Picrorhiza kurrooa* Royle *ex* Benth	0.08	1.2	50.82
*Poa alpina* L.	0.23	1.1	46.58
*Polygonum polystachyum* Wall. *ex* Meisn.	0.4	1.8	76.22
*Potentilla argyrophylla* Wall. *ex* Lehm.	0.5	2.2	93.16
*Potentilla atrosanguinea* G. Lodd. *ex* D. Don	0.3	2.1	88.93
*Potentilla fulgens* Wall. *ex* Hook	0.13	1.7	71.99
*Primula denticulata* Sm.	0.4	2.9	122.81
*Prunella vulgaris* L.	4.43	6.3	266.79
*Ranunculus hyperboreus* Rottb.	0.4	6.1	258.32
*Rumex nepalensis* Spreng.	1.19	10.8	457.35
*Salix lindleyana* Wall. *ex* Andersson	0.01	22.6	957.04
*Taraxacum officinale* F.H. Wigg	9.79	15.04	636.90
*Trachydium roylei* Lindl.	19.33	22.2	940.11
*Trifolium repens* L.	0.53	4.2	177.86
*Valeriana hardwickii* Wall.	0.04	0.5	21.17
*Viola biflora* L.	4.24	4.2	177.86

*Mean values are considered.

During the last 5 years, the tourists’ flow in the Dayara bugyal was found to be increased by 186%. Nearly 2850 tourists are found to be increasing every year. According to the linear regression predictive model, the tourists’ number may be projected to be 30,999 per year by 2025, which indicates an expected increase of 343% tourists (Supplementary Table S5). The tourists’ carrying capacity of the area was calculated to be 80,093–100,116 tourists per year (Table 2), taking into account the correction factors like rainfall, snowfall, tourists’ infrastructure, ecological parameters and socio-economic parameters. The tourists’ influx of the Dayara bugyal in 2019 was nearly 54 tourists per day. While based on our estimation, 275 tourists may be allowed for the place which reflects the necessity to promote tourism in the area.

**Table 2:** Estimation of tourists’ carrying capacity for the Dayara Bugyal (A = Available area for tourists use, Au = Area required per tourist, Rf = Daily open period / average time of visit, Cf 1 = Correction factor 1(rainfall), Cf2 = Correction factors 2 (snowfall), RCC = Real Carrying Capacity).

Total geographical area (km^2^)	Total geographical area (m^2^) (I)	Ecologically fragile area (m^2^) (II)	Available area (m^2^) (I-II)	Available area for tourism (m^2^) (A)	A/AU (A/5)	(A/AU)*Rf (Rf = 2)	Cf1	Cf2	RCC per year	RCC per day
3.94	3,940,000	12,000	3,928,000	471,360 (12% of available area)	94,272	188,544	0.59	0.72	80,093	219
				589,200 (15% of available area)	117,840	235,680	0.59	0.72	100,117	274

For the measurement of tourism activity, Hon'ble High Court Uttarakhand, Nainital, in WPPIL No.123 of 2014 order, 200 tourists per day for bugyal areas need to be followed. After analysing tourists’ data of the area, it was found under control and are not exceeding more than 200 tourists per day.

**Figure 3:**
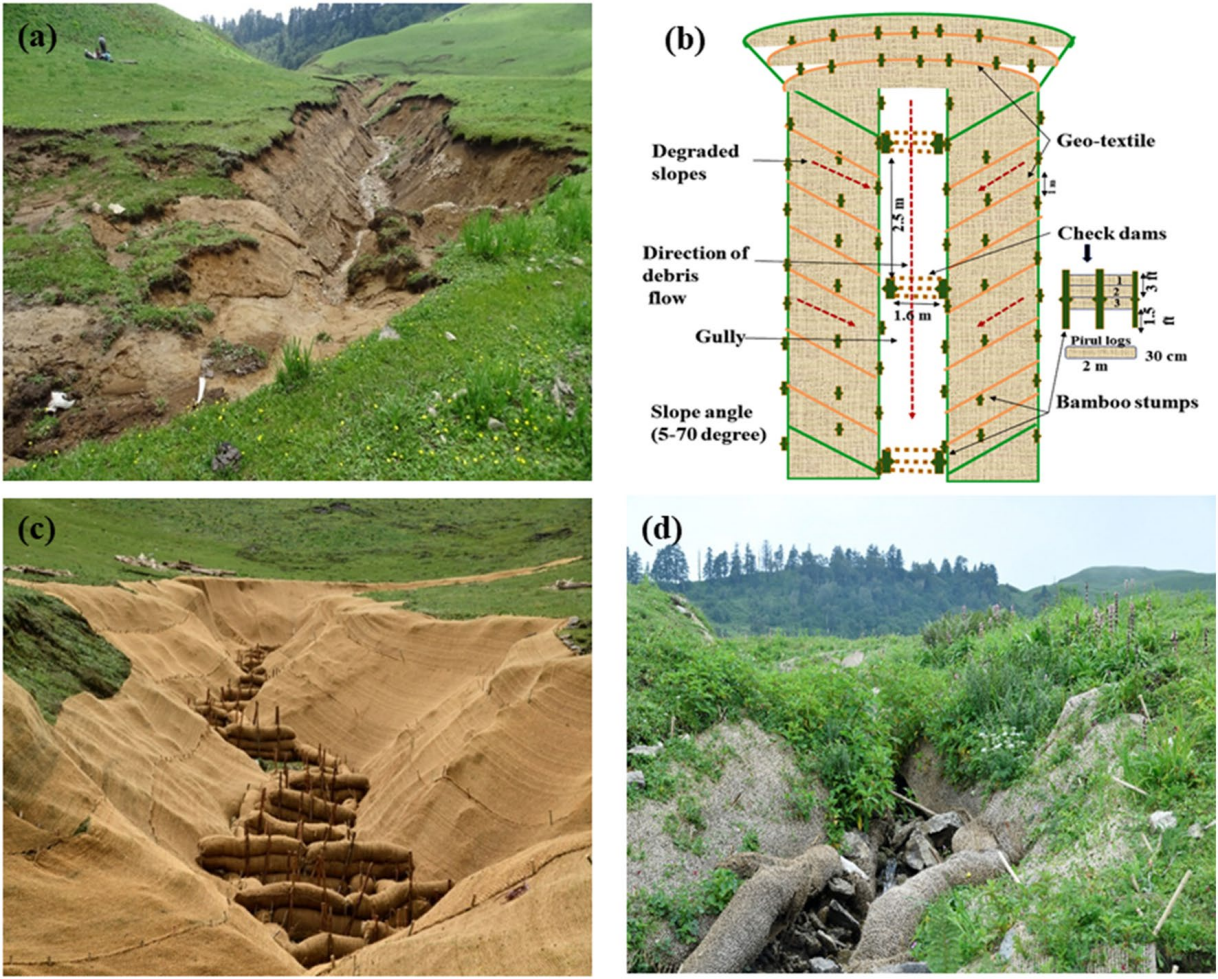
Soil erosion control techniques in the Dayara alpine pasture: (**a**) eroded gully side areas due to biotic and abiotic factors in 2018, (**b**) restoration planning details in 2019, (**c**) field implementation of the plan initiated in 2019, and (**d**) implications of one year outcome of the restoration activity since 13th October, 2020.

In view of controlling soil erosion, 170 tonne of soil was found to be arrested by the 38 check dams created among three gullies (Supplementary Table S6). The horizontal sheet erosion in highly steep sloped areas near the gullies also found to be controlled using the eco-friendly geo textile matting (Fig. 3).

### Environmental desirability (E)

**Figure 4:**
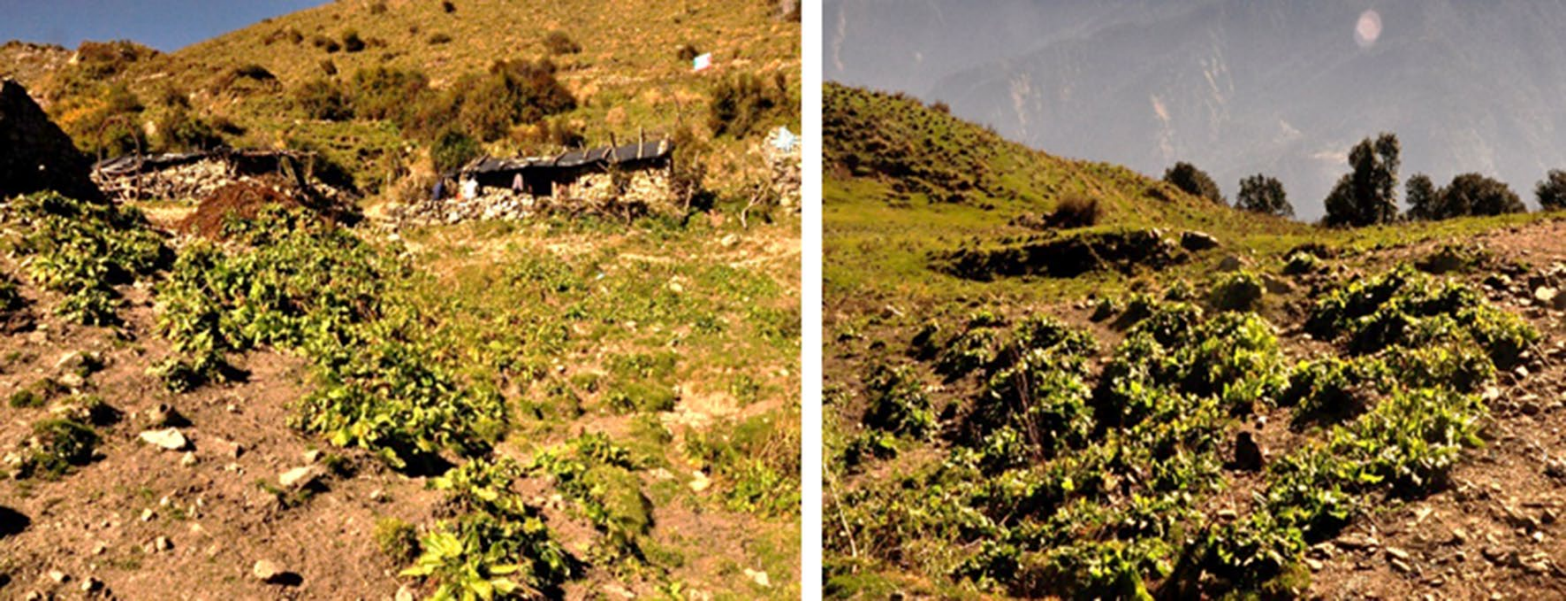
Colonization of *Rumex nepalensis* distributed in the Dayara bugyal.

Environmental desirability was measured by considering both above and below ground ecological parameters in the area. The negative influence of degradation in alpine meadow on regeneration of herbs and soil water availability has considerable impacts. The entire area was evaluated in terms of three zones, viz., geo-coir treated zone (GTZ), untreated degraded zone (UTZ) and untreated undegraded zone (R). Both soil and vegetation analyses were done in all the three respective zones to minimize the ecological effects of the restoration work. In case of above ground parameters, herbs diversity of the treated zone was found to be increased when compared with the untreated zones. The total herb density per metre square was analysed for vegetation diversity within geo-coir treated zone (GTZ), untreated degraded zone (UTZ) and untreated undegraded zone (R), respectively. Dominating plant species existing at the time of field study of the respective zone are given in Table 3. In the untreated undisturbed reference zone, dominated community was *Danthonia cachemyriana*—*Sibbaldia parviflora*-*Anemone obtusiloba D. Don*—*Achillea millefolium* community. Whereas, geo-coir treated zone was found to be dominated by *Sibbaldia parviflora*—*Taraxacum officinale—Achillea millefolium—Artemisia vestita* community. Dominance of *Danthonia cachemyriana*—*Rumex nepalensis*—*Achillea millefolium* community was found in untreated degraded zone.Colonization of *Rumex nepalensis* was found in high frequencies in different places where anthropogenic disturbance was reasonably found (Fig. 4).

**Table 3:** Dominant herbs (density/m^2^) frequency (%) and abundance among comparison in Untreated Undisturbed Zone (R), Geo-Coir Treated Zone (GTZ), and Untreated Degraded Zone (UTZ) of the Dayara bugyal.

Name of plants	Density (individual/m^2^) (mean)	Frequency (%)	Abundance
**Untreated Undisturbed Zone (R)**			
*Achillea millefolium* L.	1.40	73.33	1.91
*Allium humile* Kunth	0.70	36.67	1.91
*Anaphalis contorta* (D. Don) Hook. f.	0.77	43.33	1.77
*Anemone obtusiloba* D. Don	1.47	70.00	2.10
*Arctium lappa* L.	0.40	26.67	1.50
*Artemisia vestita* Wall. *ex* Besser	1.13	63.33	1.79
*Bistorta affinis* (D. Don) Greene	0.60	40.00	1.50
*Cyananthus lobatus* Wall. *ex* Royle	0.87	50.00	1.73
*Danthonia cachemyriana* Jaub. & Spach	1.70	90.00	1.89
*Epilobium laxum* Royle	0.67	43.33	1.54
*Geranium wallichianum* D. Don *ex* Sweet	0.37	23.33	1.57
*Kobresia nepalensis* (Nees) Kk.	0.90	33.33	2.70
*Morina longifolia* Wall. *ex* DC.	0.43	23.33	1.86
*Poa alpina* L.	0.80	46.67	1.71
*Potentilla argyrophylla* Wall. *ex* Lehm.	0.80	46.67	1.71
*Potentilla atrosanguinea* G. Lodd. *ex* D. Don	1.13	63.33	1.79
*Prunella vulgaris* L.	1.27	53.33	2.38
*Sibbaldia parviflora* Willd.	1.93	83.33	2.32
*Tanacetum longifolium* Wall. *ex* DC.	0.73	43.33	1.69
*Taraxacum officinale* F.H. Wigg	1.23	53.33	2.31
*Viola biflora* L.	0.47	36.67	1.27
**Geo- Coir Treated Zone (GTZ)**			
*Achillea millefolium* L.	0.97	56.67	1.71
*Allium humile* Kunth	0.23	23.33	1.00
*Anemone obtusiloba* D. Don	0.50	40.00	1.25
*Artemisia vestita* Wall. *ex* Besser	0.93	53.33	1.75
*Danthonia cachemyriana* Jaub. & Spach	0.80	60.00	1.33
*Epilobium laxum* Royle	0.60	46.67	1.29
*Impatiens sulcata* Wall.	0.30	23.33	1.29
*Morina longifolia* Wall. ex DC.	0.13	13.33	1.00
*Poa alpina* L.	0.43	30.00	1.44
*Polygonum polystachyum* Wall. ex Meisn.	0.50	36.67	1.36
*Potentilla argyrophylla* Wall. *ex* Lehm.	0.57	40.00	1.42
*Potentilla atrosanguinea* G. Lodd. *ex* D. Don	0.60	40.00	1.50
*Prunella vulgaris* L.	0.77	40.00	1.92
*Senecio chrysanthemoides* DC.	0.60	40.00	1.50
*Sibbaldia parviflora* Willd.	1.23	73.33	1.68
*Tanacetum longifolium* Wall. *ex* DC.	0.27	20.00	1.33
*Taraxacum officinale* F.H. Wigg	1.13	56.67	2.00
**Untreated Degraded Zone (UTZ)**			
*Achillea millefolium* L.	0.43	30.00	1.44
*Anemone obtusiloba* D. Don	0.30	20.00	1.50
*Artemisia vestita* Wall. *ex* Besser	0.40	33.33	1.20
*Danthonia cachemyriana* Jaub. & Spach	0.63	33.33	1.90
*Epilobium laxum* Royle	0.27	20.00	1.33
*Potentilla argyrophylla* Wall. *ex* Lehm.	0.40	23.33	1.71
*Rumex nepalensis* Spreng.	0.57	33.33	1.70
*Taraxacum officinale* F.H. Wigg	0.40	30.00	1.33
*Polygonum polystachyum* Wall. *ex* Meisn.	0.30	23.33	1.29

In case of below ground in the GTZ parameters, like water holding capacity of soil was found to be 50% which is 3% higher than the untreated degraded site (UTZ), i.e., 47%, within a span of one year after treatment. The water holding capacity of undisturbed soil was 57% which is expected to be increased in successive years. Increase in water holding capacity will increase soil moisture content (SMC) which will help plants to grow more and during later stages it may overcome negative soil water potential (SWP). SWP is a fundamental hydrological variable that indicates soil water status and is linked with plant physiology. The growth of vegetation in alpine regions is generally impeded by rigorous hydrothermal conditions and limitations in soil nutrient. Rise in nitrogen, total potassium, and total phosphorus was also examined in the GTZ comparing with the UTZ (Table 4).

**Table 4:** Chemical characteristics of the different soil zones (upto 30 cm depth) from R, GTZ and UTZ in the Dayara bugyal (n = 3 per investigation zone; 50 random soil cores per replicate for soil analysis ; 30 randomly placed quadrates of 1 × 1 m in 5-50 m in triplicate per investigation zone).

Variables	Investigation zone		
	Untreated Un degraded zone (R) *	Geo-coir Treated Zone (GTZ)*	Untreated Degraded Zone (UTZ)*
**Vegetation profile**			
Total herb density / m^2^	19.77 ± 0.04	10.56 ± 0.02	3.7 ± 0.05
**Soil chemical profile**			
pH	5.15 ± 0.24	4.96 ± 0.28	4.69 ± 0.21
OC (%)	6.64 ± 0.26	4.83 ± 0.04	3.76 ± 0.14
N (%)	0.133 ± 0.01	0.09 ± 0.03	0.047 ± 0.02
P (%)	0.35 ± 0.04	0.29 ± 0.03	0.14 ± 0.01
K (%)	0.81 ± 0.02	0.78 ± 0.04	0.26 ± 0.02
WHC (%)	56.96 ± 0.13	49.85 ± 0.13	47.76 ± 0.59

*(mean ± standard error).

### Socio-economic feasibility (SE)

**Figure 5:**
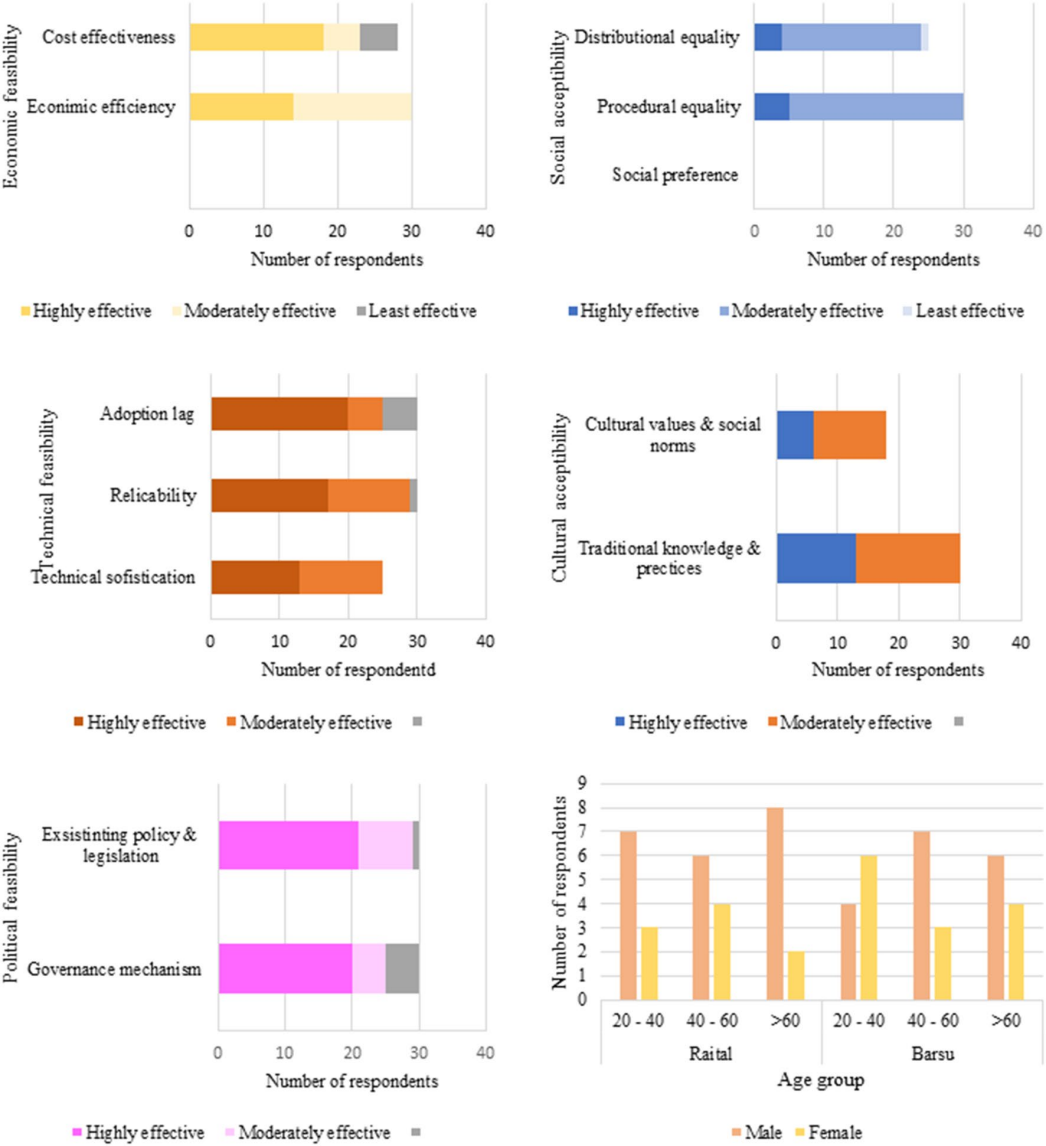
Socio-economic responses by the local stakeholders regarding feasibility assessment criteria related to the Dayara bugyal restoration.

Socio-economic feasibility of the work was evaluated by village survey conducted in two adjacent villages within a periphery of the Dayara bugyal, Barsu (2258 m) and Raithal (2180 m). The detailed outline of the respondents regarding the selected socioeconomic parameters are depicted in Fig. 5 (Supplementary Table S7, S8). Locally available materials like pine needles, bamboo and involving villagers as workers in view of generating local employment have not only resulted in reduction of cost by about 20% but also has generated direct and alternate livelihood opportunities for 700 households. During restoration planning, Biodiversity Management Committees (BMCs) of nearest these two villages were involved for ensuring sustainability in alpine and sub-alpine meadows and their downslope located villages. Regular meetings conducted with the villagers since 2018 have shown that there is a significant reduction in the number of unattended cattle. Innovative techniques using biodegradable coir-geotextile, locally available pine needles and bamboo have thus emerged out as an economical and sustainable alternative to treat degraded meadows and has resulted in more effective regeneration of vegetation. Being labour intensive, this has not only generated livelihood alternatives to the local people but also has reduced the fire incidences due to Pine forests in the lower altitude of the district. Being technically simple, the treatment works were carried out by the villagers without any inherited knowledge and experience according to Divisional Forest Officer (DFO), Uttarkashi. The technique therefore has emerged as one of the most suitable method of treatment of degraded alpine meadows in the Himalayan region which was degraded more naturally due to gully erosion than anthropogenic pressures.

### Index score

**Figure 6:**
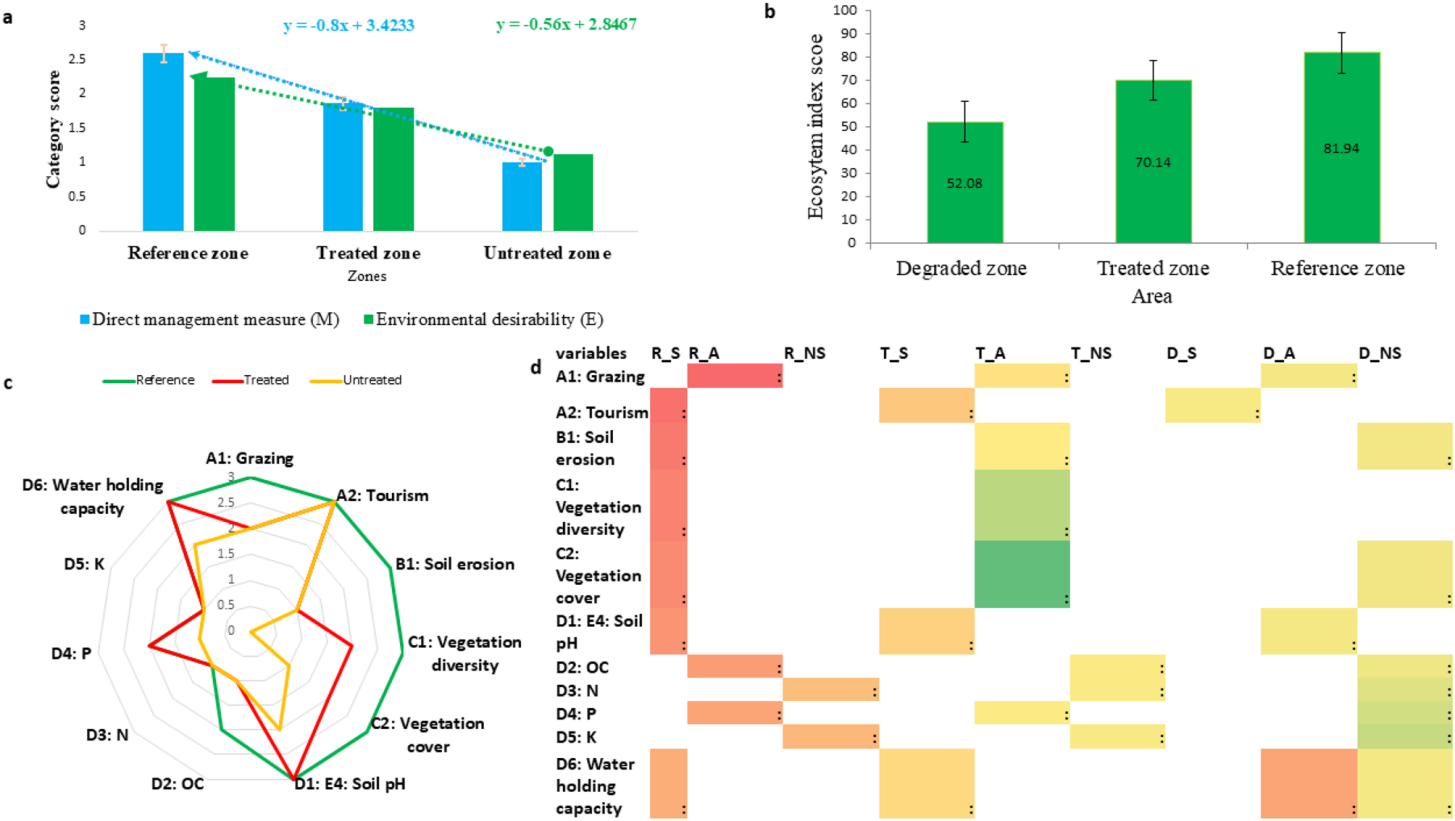
Evaluation of different components under restoration evaluation index; (**a**) category comparison between different zones, (**b**) ecosystem index score comparison between restore, reference and degraded zones, (**c**) evaluation of individual variables for their effectiveness in different zones, and (**d**) ecological profile of different zones (R_S = satisfactory for reference zone, R_A = average for reference zone, R_NS = not satisfactory for reference zone, similarly T = treated zone, D = degraded zone).

**Figure 7:**
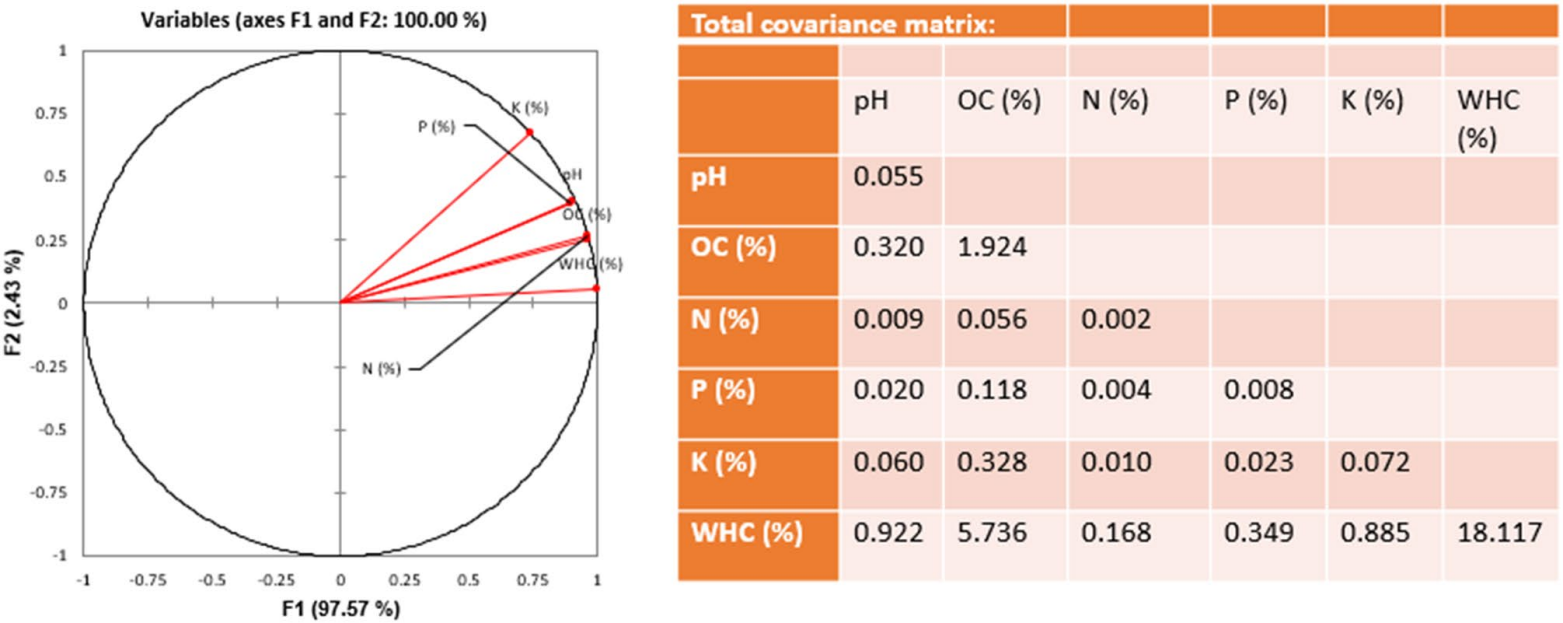
Discriminant function analysis of (**a**) direct field evaluated values, and (**b**) their covariance matrix.

All individual variables of the first two categories were indexed according to the defined scale formulated on the basis of available literature and expert opinion (Table 5). In case of the last category, scaling was done directly by considering individual opinion for scoring information. The restoration evolution index of the entire work was calculated as 69.31 (Supplementary Table S9). Among the three categories of the index, changes governed by management measures (0.8) used in the work were found to be more effective than the environmental changes (0.56). Ecosystem index scores were determined among references, degraded and restored zones applying one way ANOVA analysis wherein two categories viz., direct management measure (M) and environmental desirability (E) were used. The average ecosystem index score for treated zones was 72% (± 3.78 standard error [SE]) which was significantly higher than the average score of degraded zones 55.61% (± 4.79) but lower than the average score of reference zones 88% (± 3.17) (one way ANOVA test, p = 0.003, Fig. 6b). The highest variation was observed in a degraded zone (68%), followed by treated zone (42%) and reference zone (30%). Now, considering the individual variables, the score of water holding capacity and soil pH of treated land were found to be in most comfortable zone comparing with the non-degraded zone as a reference. Tourism activity was not found to be responsible for the degradation as the scores were almost equal in reference and degraded zones. From the hit map analysis of the variables, it was made clear that soil chemical properties like OC, N and K were not found to be in good agreement in case of the treated land (Fig. 6). Direct field values of the variables considered under ecological category were further evaluated in three respective zones by discriminate function analysis. The results revealed two significant functions, factor 1 accounted for 97% of the explained variance, and factor 2 accounted for 2% of the explained variance. All individual variables of soil were found to be strongly correlated across the three zones with little low values in case of P and K (Fig. 7).

**Table 5:** Scoring criteria for calculation of restoration evaluation index.

	Satisfactory	Average	Not Satisfactory	References
**Soil chemical properties**				
pH	4.7–5.3	4–4.7	< 4.0	17, 18, 19
OC (%)	> 7.5	5.0–7.5	< 5.0	
N (%)	> 0.75	0.50–0.75	< 0.50	
P (%)	> 0.50	0.25–0.50	< 0.25	
K (%)	> 2	2–1	< 1	
Water holding capacity (%)	50–60	40–50	< 40	
**Vegetation index**				
Vegetation cover (%)	> 60	30–60	< 30	Based on field observation
**Management measures**				
Grazing control	< 285	285 -857	> 857	Based on field observation, experts’ opinion and survey, Order of Hon'ble High court, Uttarakhand (WPPIL No.123 of 2014 ), 21, 22
Tourists’ control	< 65	65–200	> 200	
Erosion control (t h^-1^y^-1^)	< 5	5–35	> 35

## Discussion

The alpine pastures in the Himalaya provide a wide range of ecosystem services such as carbon sequestration, water storage and provisioning, maintaining biodiversity, food security and livelihoods. But current days, these ecological treasure houses are facing degradation threats like upcoming invasive species, inappropriate management and development policies, soil erosion, extraction of medicinal plants, overgrazing and climate change^23^. Therefore, these areas require not only the protection of native vegetation but also its restoration wherever necessary. Eco-restoration is an attractive strategy in this regard that will provide the ecological and socioeconomic benefits like expanding coverage and connectivity of the remaining native vegetation; increasing the flow of ecosystem goods and services (e.g., water, food, biological control, pollination, grazing livestock products, timber and non-timber forest products, climate regulation, control and mitigation of erosion and floods); and creation of social and economic development opportunities for rural community^24^. On the other hand, implementing eco-restoration is a challenging work for these regions. The outcomes of the present work, depicts a clear eco-restoration and its evaluation framework for degraded high altitude alpine pastures of the Himalayan region. The degradation parameters considered for the present work was found in harmony with other affected areas of the Himalayan Region. Like, overgrazing is reported for the areas of high altitude grasslands of Kashmir, Tungnath, Garhwal in the Indian Himalaya, Sagarmatha (Mt. Everest) National Park, Nepal and China^25–28^, and other places of the Himalaya. In case of the Dayara bugyal, local livestock populations were found to be increased at a rate of 33 CU per year and also evaluated to be more responsible for overgrazing. Migratory livestock unit was found to be controlled after the restoration phase. Overgrazing is strongly associated with soil cracking and vegetation disturbance, important processes and features of degradation in alpine ecosystems^29^. Shifting of grazing land from Dayara towards Lambidhar was an excellent management measure in this aspect, where more control measures are needed. According to our calculations, if 445 CU will be deducted from the local livestock from Dayara, grazing will be under carrying capacity. Therefore, the grazing pattern of 118 migratory CU (76 horse/mule and 18 sheep/goat, already controlled) and 318 local CU (30 horse/mule and 187 sheep/goat, need to be controlled) is recommended for the area. Rest of the animals may be shifted to some other areas like Lambidhar, Devkund, and Suriyatop. One check post for checking the livestock should be made in this regard.

Further, in alpine region, tourism is also reported to reflect impacts like reduction in litter biomass, soil nutrient supply and soil enzyme activities, generation of solid waste, thus adversely affect the whole plant-soil system^30^. On the other hand, tourism sector is considered as growth engine for the future development of the Indian Himalayan Region^31^. In Uttarakhand, due to lack of attentive strategies, and the existing policy framework, incongruous practice of tourism, unplanned developmental activities, and the massive inflow of visitors are some of the key factors adversely affecting the sustainable tourism development^32^. The results of tourist carrying capacity inflow of 274 tourists per day for the area was also in compliance with Hon'ble High court, Uttarakhand (WPPIL No.123 of 2014) where 200 tourists per day are allowed to visit on alike locations.

Tourism activity of the study area was found to be below carrying capacity and further eco-tourism may be promoted in the area which will also have socioeconomic impact for the two adjacent villages. Promotion of homestays for tourists, training of local people as tour guides and bird watching are suggested for the same. Some base framework suggested for a community-based rural tourism development include activities followed in Darap and Pastanga villages in Sikkim and Dhanolti Eco-park in Uttarakhand^33,34^. A village level committee like Dayara Ecotourism Development Committee (DEDC) may be formed in the upcoming phases, consisting of 20 members (10 from Raithal, 10 from Barsu villages) representing 50% women members. The main role of the committee will be to manage the tourists’ influx, conserve forest and ecosystem, dispose of and recycle garbage, and collect fees for various amenities as provided to the tourists. A small nature interpretation and learning centre may be also established in Barsu village, by engaging localities in Eco-huts. The role of the centre will be conducting amusement facilities like flying fox and burma bridge, plantation of memory saplings, and nature trails with yoga for the tourists. Localities may be made aware of the activities, by way of nature learning and interpretation training provided by GBP-NIHE, Kosi-Katarmal, Almora, Uttarakhand, India. Further, practices like packaging with less waste prone materials such as decomposable wrappers, establishment of a waste dealer centre (Could one earn 25 paise per bottle upon submission, if sold to a dealer) are also adopted in the area for the management of solid waste^35^.

Soil erosion of the area was found to be controlled at the rate of 0.5 tonne per hectare per year. The newly introduced geo-coir matting approach was found to be successful in this aspect. The geo-coir matting technique was successful for controlling soil erosion in the Amachal watershed in Trivandrum District in the Western Ghat region of Kerala, India^36^. But its use in such a high-altitude alpine in the present work is first time found to be successful. Further, use of dry pine needles (*Piruls*) in the construction of cheek-dams was also found to be an effective strategy as these are highly inflammable because of presence of turpentine oil in them and cause hunting forest fire incidences in the north western Himalaya during summer^37^.

Thereafter, environmental desirability in the treated area was found to have improved vegetation and soil profile. The total herb density in per metre square of the treated area indicates good values. But colonization of *Rumex nepalensis* was found in different places where anthropogenic disturbance was frequently found. This species has good forage value, higher crude protein (CP) and digestibility for cattle. On the other hand, it has become dominant and outcompete desirable pasture species and degrade pasture quality. It regenerates from tap roots and establishes quickly as seedlings. Once established, tough tap roots become difficult to remove and are not readily damaged by tillage^38^. Therefore, manual removal of *Rumex nepalensis* at the shepherd camping (30°50′3.48"N, 78°34′2.21"E to 30°50′0.49"N, 78°34′2.76"E, 3348 m) is recommended to stop the future spread of this difficult weed in the pasture area. Small plots of 10 m^2^ can be considered for this purpose stating from the camping sites. Simultaneously, plantation activity should be done to stop the surface soil erosion. Herbs like *Trifolium repens*, *Carex setosa* and *Dolomiaea macrocephala* are recommended for this purpose. In below ground, water holding property of soiland soil nutrients were also found to increase in the treated area. Soil pH of the treated area was also found to be undisturbed. Pine needles are a matter of concern as these were introduced in the bugyal area during the restoration work. Soil ‘C’ and ‘N’ storage and other physiochemical components were found to be increased in the treated zone comparing with the untreated zone. This will ultimately increase the above ground biomass of the area^39^. Reduction of the grazing activity could be one of the reasons behind it. On the other hand, during upcoming 10 years, exclusion of the grazing activity may reduce the vegetation diversity of the area and may allow the vegetation to be dominated by a few species with strong colonization abilities^40^.

The socio-economic parameters used in the present study was the field implementation of the conceptual framework designed by Pandit et al. (2020) for land degradation and restoration responses for improved planning and decision-making considering 141 current articles in this context. Although the framework was for forest land degradation assessment, but the parameters also strongly correlate with the pasture area of the current work. The results of the present work revealed good success rate and popularity of the work among the localities as well as in Government departments. Community involvement is a vital parameter behind the success of any work. Like Community Forest User Groups in Nepal benefited nearly 2.908 million households by 2018 and also managed total of 2.238 million ha of forests^41^. The ecological index of the treated zones was found to be increased in comparison with the degraded zone which indicated improving ecological profile of the area. Direct management measures like control in grazing and tourism activity along with geo-textile matting and check dams for erosion control are found to have more impact in this aspect.

Therefore, the eco-restoration strategies and their evaluation model of the present work, could have its future avenue for successful restoration practice. The planning of geo-coir mats and *pirul* check dams for the soil erosion control may be further used for restoration of other high-altitude regions prone to erosion. The grazing pattern and herbs colonization controlling strategies, tourism management strategies as suggested in the work are also useful for other degraded pasture lands facing the same problem in high altitude degraded alpine pastures of the Indian Himalayan Region as well as in other mountain alpine and sub-alpine ecosystems.

## Methods

### Restoration response evaluation

Multi-criteria response analysis has been a crucial part of restoration evaluation work as a proper practical achievement which always includes multiple objectives defined by diverse stakeholders. In current work, a new framework was designed for restoration response evaluation by assessing three categories, direct management measure (M), environmental desirability (E) and socio-economic feasibility (SE). In total, 9 sub-categories and 22 individual variables were considered for evaluation of the present work^41^ (Table 6).

**Table 6:** Response evaluation parameters.

Categories	Sub- categories	Individual variable
Direct management measure (M)	M1: Anthropogenic (A)	A1: Grazing
		A2: Tourism
	M2: Natural (B)	B1: Soil erosion
Environmental desirability (E)	E1: Above ground (C)	C1: Vegetation diversity
		C2: Vegetation cover
	E2: Below ground (D)	D1: E4: Soil pH
		D2: Organic carbon (OC)
		D3: Total Nitrogen (N)
		D4: Total Phosphorus (P)
		D5: Total Potassium (K)
		D6: Water holding capacity
Socio-economic feasibility (SE)	SE1: Economic feasibility (E)	E1: Cost-effectiveness
		E2: Economic efficiency
	SE2: Social acceptability (F)	F1: Procedural equity
		F2: Social preference
	SE3: Technical feasibility (G)	G1: Adoption lag
		G2: Replicability of the response
		G3: Technical sophistication
	SE4: Cultural acceptability (H)	H1: Cultural values
		H2: Social norms
	SE5: Political feasibility (I)	I1: Policy/legislation
		I2: Governance mechanism

### Direct management measure (M) evaluation

During the starting phase of the work, excessive grazing, uncontrolled tourism and continuous soil erosion were identified as major drivers behind the degradation of Dayara bugyal. Therefore, in the first category of the evaluation work, direct management measures to control the above-mentioned activities were analysed. The disturbances were controlled by managing both anthropogenic (M1) and natural (M2) processes. Under anthropogenic control process, grazing (A1) and tourism (A2) control activities were measured and soil erosion (B1) control activities was considered under natural control sub-category.

### Livestock carrying capacity

Livestock carrying capacity of the pastureland was a measure for proper control of the estimation of grazing capacity. Forage yield of the area was calculated by considering the shoot production of 10 dominant palatable species of the area. Sample plant materials at the end of the growing season, were oven-dried at 80 $^\circ$C till it reached at constant weight and then weighed in the laboratory. Thereafter, density of individual plant was measured by laying 30 quadrates of 1 × 1 m randomly placed within 50 × 50 m grid in the herb community (Eq. 1)^42^. Total 80 grids were sampled for analysis of 40 hectare degraded grazing land of the Dayara alpine pasture from the Papad Gad and Swari Gad area. Thereafter, 10 dominant palatable species covering ~ 33% of the total dry weight of the palatable and unpalatable species were considered for forage production calculation. Peak biomass was calculated by summing up the peak biomass of each individual to get the forage yield (Eq. 2). Finally, standard dry forage yield and proper rangeland carrying capacity was calculated by using Eqs. (3) and (4)^43^ as follows.

1 $${\text{Density }} = \frac{{\text{Total number of individuals of a species in all quadrates}}}{{\text{Total number of quadrates laid}}}$$

2 $$Y = Y{\text{p}} \times A$$

where,* Y* = forage yield in a certain area (kg), *Y*p i = forage yield per unit area (kg/km^2^), *A* = land area of rangeland (km^2^) (i.e., total grazing area of the Dayara occupies 3.235 km^2^).

3 $$F = \mathop \sum \limits_{i = 1}^{n} Y_{{\text{i}}} \times {\text{ U}}_{{\text{i}}} \times {\text{ C}}_{{\text{i}}}$$

where F = yield of standard dry forage (kg), *Y*i = forage yield (kg), *U*i = utilizable rate (%), *C*i = conversion coefficient.

Utilization rate 50% and conversion coefficient 1 for meadow was considered for current work^43^ .

4 $$Cc = \frac{{\text{F}}}{{{\text{I}} \times {\text{D}}}}$$

where, *C*c = proper livestock numbers that meadow can bear, F = yield of standard dry forage (kg), I = daily intake for an animal unit (7.5 kg/day, Table 7)*, D = Grazing days (May to September, 153 days).

**Table 7:** Animal unit and forage requirement.

Animals	Avg. body weight (kg)	Forage requirement (dry matter in kg/day)	Animal Unit
Adult milking cow	250	7.5	1
Horse / mule	312.5	9.38	1.18
Goat	35.78	1.07	0.23
Sheep	32.74	0.98	0.22

*One animal consumes 3% of its body weight as dry forage^44^. Animal unit conversion was done after Rawat (2020)^45^.

#### Tourists’ Carrying Capacity (TCC)

The general formula of carrying capacity assessment for protected areas was first proposed by Cifuentes (1992), which was further applied in different fields^46^. The approach is to establish the capacity of an area for maximum visits based on existing physical, biological, and management conditions through the physical carrying capacity (PCC), and real carrying capacity (RCC). TCC is divided into the following levels:

#### Physical Carrying Capacity (PCC)

The PCC is the maximum number of tourists that can physically accommodate into or onto a specific area, over a particular time. The PCC (Eq. 5) may be estimated as follows:

5 $${\text{PCC }} = {\text{ A}}/{\text{Au}} \times {\text{ Rf}}$$

where, PCC = physical carrying capacity; A = Available area for tourists use ; 15%-18% area of the total geographical area is considered for the present work according to the expert opinion and URDPFI guidelines for hill towns^47^.

Au = Area required per tourist; in general, it is considered 3 m^2^. However in the present work, 5 m^2^ area is considered for one person based on nature of the area is relatively more sensitive to degradation.

Rf = Daily open period / average time of visit.

Average opening time = 6 h (according to the field survey, tourists like timing for a day visit between 9 AM to 3 PM), time required by one tourist to visit the Dayara bugyal = 3 h.

Rf = 6 h/3 h = 2.

Real Carrying Capacity (RCC) (Eq. 6)

Maximum permissible number of tourists to a specific site could be determined once the Correction factors (CF) becomes possible to derive out of the particular characteristics of the site. CF is applied to the PCC as follows.

6 $${\text{RCC }} = {\text{ PCC }} \times \, \left( {{\text{Cf1}} \times {\text{ Cf2}} \times {\text{ Cf3}} \times {\text{ Cf4}} \times \cdots {\text{Cfn}}} \right)$$

where RCC = Real Carrying Capacity, PCC = Physical Carrying Capacity, Cf = Correction factors.

Correction factors are calculated using the following formula.

$${\text{Cfx }} = { 1 }{-}{\text{ Lmx }}/{\text{ Tmx}}$$

where Cfx = Correction factors of variable x, Lmx = Limiting magnitude of variable x, Tmx = Total magnitude of variable x.

Tourism is dependent on nature. In the present work, number of days with heavy rain (> 250 mm per day) and snowfall (> 8 cm per day) were considered as limiting variables that control tourism for the area. The calculations were done by analyzing the rainfall and snowfall data from 2017 to 2019 considering March to November as rainfall months and December to February as snowfall months. Total numbers of days in the months were considered as total variables (Tmx) and the days with heavy rain/snow fall were considered as limiting variables (Lmx). For example, during 2017 to 2019 total number of days from March to November were 909 days (Tmx) and in 369 heavy rainfall occurred, therefore, Cf1 was 0.59 (1—Lmx/Tmx). Similarly, during this time heavy snowfall occurred for 51 days out of 186 days, and Cf2 was 0.72.

### Measurement of soil erosion control

Eco-friendly bio-degradable coir geo-textile (9000 sq m) purchased from Coir Board of India, locally available pine needle (240 tonne) along with bamboo were roped in, to create a series of check dams and channels to control soil erosion, gully formation and vegetation loss. Prior to commencement, the leveling of uneven surfaces was done before laying the coir geo-textile. The open degraded sites in different patches of the bugyal, the eroded lateral sites of the gullies were then covered with geo-textile to control soil erosion. Total 38 check dams in the Swari Gad area were examined for draining soil holding capacity. After one year of the treatment, total mass of debris stored by each check dams was evaluated using core density method. The core density of bulk soil in each check dam was determined in triplicates, using an iron core of 2.5 cm radius and 30 cm height. The mass of draining soil checked by each check dam was calculated as under^48^:

$${\text{Md }} = {\text{ V }} \times \, \rho {\text{b}}$$

where, Md = The mass of debris in each check dam, V = Volume of check dam, ρb = Mean core density of bulk of soil in each check dam.

### Environmental desirability (E) assessment

In this part, environmental desirability, the direct ecological outputs of the work were considered under this category, as habitat enhancement is the most crucial component of the activity. The sub-component considered under the category included vegetation structure (vegetation diversity, vegetation cover) and ecological progress (soil chemical properties)^20^. Vegetation sampling was done by considering 30 randomly placed quadrates of 1 × 1 m inside 9 sample plots of 5-50 m along three different zones of the treated water channel areas using vertical belt transact method^49^. The zones were: (i) geo-coir treated area, (ii) untreated degraded area, (iii) reference untreated non-degraded area along with both sides of the water channels wherein total vegetation density (Eq. 1) was analysed following the methodology of Misra (1968) and Mueller-Dombois & Ellenberg (1974)^50,51^.

### Soil sampling

Soil samples (30 cm depth) were collected from the experimental site in triplicates using random sampling method from all the three investigation zones, namely, Untreated undegraded zone (R), Geo-coir Treated Zone (GTZ) and Untreated Degraded Zone (UTZ). Fresh samples were taken from each plot (50 random soil cores per replicate per investigation zones) and were mixed thoroughly as one composite sample for further study. Here, it is to mention that utmost care was taken to collect each replicate as composite soil sample to appropriately represent the investigation zones of varied topography. Hence, total 9 soil samples (3 samples per investigation zones) were collected to determine its physico-chemical characteristics. After collection, the soil samples were preserved in a portable storage box and transported to the lab immediately. After air drying and grinding, it was passed through 2-mm sieve, and selected soil properties viz. soil organic carbon (SOC) (%), soil pH, total nitrogen (N), phosphorus (P), potassium (K) contents (%), and water holding capacity (WHC) (%) were determined.

### Soil physico-chemical analysis

The SOC content in soil was determined by wet oxidation method using K_2_Cr_2_O_7_^52^. The soil pH was measured with a suspension of soil in water at a 1:2.5 (soil : water) soil-to-solution ratio using a glass electrode. Calibration of the pH meter was done with the help of two buffer solutions of pH 7.0 and 9.2^53^. The WHC of the soil was determined by measuring the ratio of total water in the wet soil to the weight of the air-dried soil using a Keen– Rackzowski box^54^. Total N was analysed following the micro Kjeldahl method^55^. Total phosphorous (TP) was determined using the HClO_4_-H_2_SO_4_ method^56^ and total potassium (TK) was measured by Flame Photometer (NaOH melting)^57^.

### Socio-economic feasibility (SE) assessment

To investigate the opinion of local residents about the restoration initiative, village survey was conducted in two adjacent villages of the Dayara bugyal, Barsu (2232 m) and Raithal (2258 m). Participants had to indicate the degree of the work in above mentioned three scales (M, E and SE). The questionnaire comprising of questions covered perception about the above discussed six categories (Supplementary S10). Total 60 respondents from different households were randomly selected from each village. The sample consisted of villagers as well as administrative staff. The informants were randomly chosen across 3 different age groups, 20–40, 40–60 and > 60 year^58^. Economic feasibility was the first class and parameters considered under this category included cost-effectiveness of the material used, economic efficiency, i.e., benefit–cost ratio and economic impact of the generated income. In addition, social acceptability is the next category, where two sub-parameters were considered, procedural equity (inclusivity and participatory) in response to planning and designing and social preference that covers over current practices, access to resources and services. In the fourth category, technical feasibility was considered which included three subcategories. Adoption lag means waiting period required to adopt the response, replicability of the response and technical sophistication associated with response. In sixth category, cultural acceptability was considered to deal with alignment of the work with cultural, spiritual and aesthetic heritage values, beliefs and social norms and use of traditional (indigenous and local) knowledge and practices. In the last category, political feasibility was considered, where existing policy/legislation and governance mechanism (clarity on roles/responsibilities of stakeholders) was analysed. Each restoration response is ranked using a relative effectiveness or performance rating scale of low (L), moderate (M), or high (H). These effectiveness response ratings for each sub-criterion also reflect no (or minimal), some (or moderate) and major (or substantial) improvement, respectively, relative to the initial condition (pre-response).

### Index score calculation

**Figure 8:**
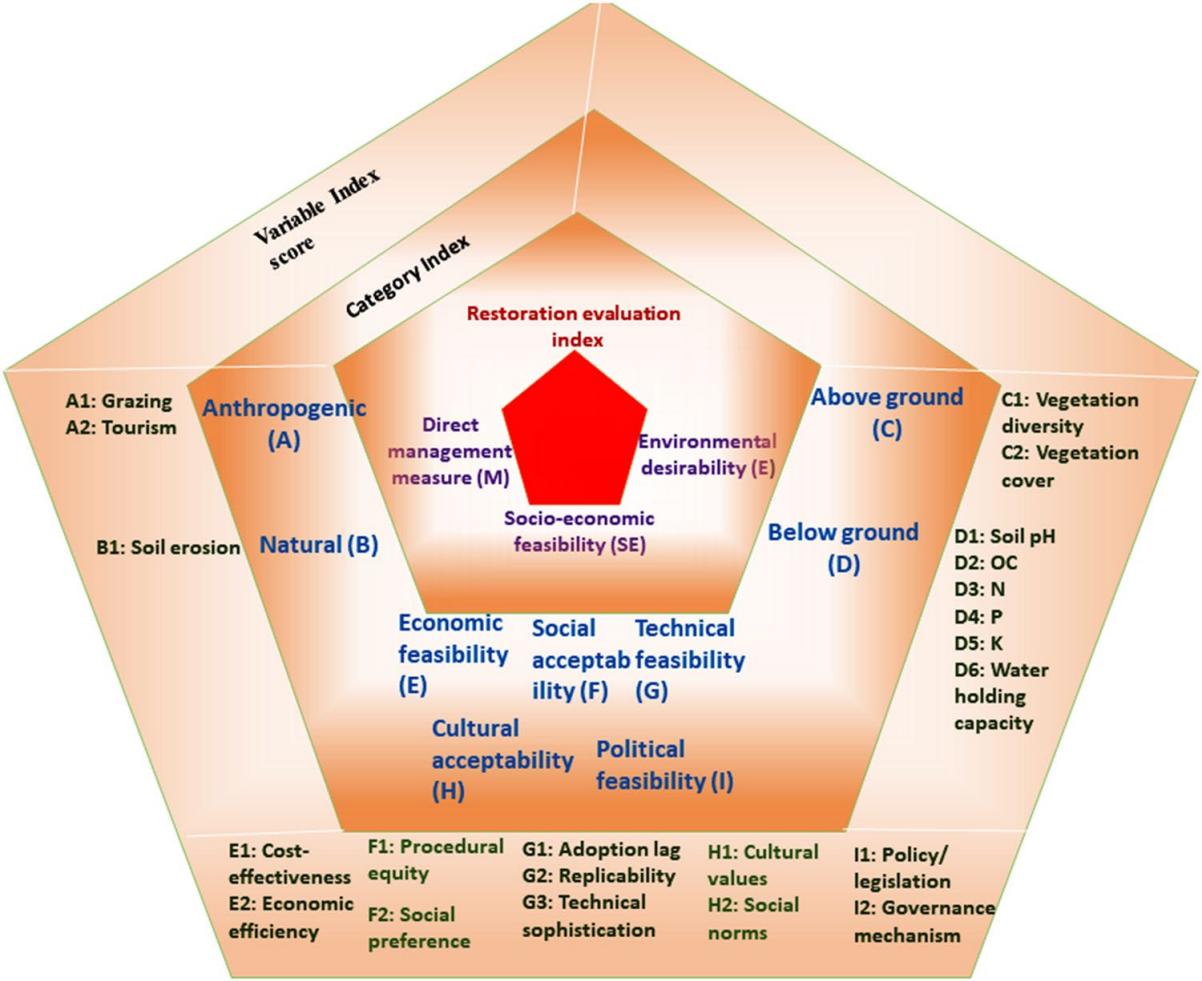
Detailed outline of the scoring process applied for restoration evaluation index calculation for the Dayara bugyal.

Restoration success index was calculated, by considering three categories, viz., direct management measure (M), environmental desirability (E), and socio-economic feasibility (SE). In the first scoring part, all the 22 individual variables were evaluated for calculation of “variable index”, by assigning index score between 0 and 3, where 0 rated for ‘not satisfactory’ and 3 rated for ‘satisfactory’. For first two categories, i.e., direct management measure (M), environmental desirability (E), and direct field values were considered. The last category, socio-economic feasibility was indexed depending on village questionnaire survey. The second score “category index” was calculated by adding all variable index and divided by number of independent variables within that category. Finally, the “restoration evaluation index” was evaluated by summing all category scores, dividing by the maximum possible score (16) and multiplying by 100^59^ (Fig. 8). Ecosystem differences between reference, degraded and restored sites category and ecosystem index scores were determined using unpaired one way ANOVA by using categories viz., direct management measure (M) and environmental desirability (E). To estimate the most affected variable between references, degraded and restored sites, discriminant function analysis (DFA) was carried out, using the field values of all measured independent variables under second category.

## Supplementary Information

Supplementary Information.

## Data Availability

Initially, the digital elevation model data used in the study area map which is available in ALOS PALSAR – Radiometric Terrain Correction section of NASA earth science data (https://search.asf.alaska.edu/). The shape file data used for Uttarakhand map is available at DIVA-GIS (http://www.diva-gis.org/). Datasets used from different climatic analysis are available at CRU (https://sites.uea.ac.uk/cru/data). All other data are available either in the main text or as supplementary materials. Nomenclatures of the plants were validated from the book, ‘Flora of Gangotri National Park, Western Himalaya’, Botanical Survey of India^60^.
