# Supplementary material for: Dayara bugyal restoration model in the alpine and subalpine region of the Central Himalaya: a step toward minimizing the impacts
Source: Sci Rep. 2021 Aug 16;11:16547. doi: 10.1038/s41598-021-95472-y (PMC8367960; doi:10.1038/s41598-021-95472-y)
Supplement: Supplementary file 1 — Supplementary Information. [file 41598_2021_95472_MOESM1_ESM.pdf]

Dayara Bugyal Restoration Model in the Alpine and Subalpine Region of the Central Himalaya: A Step toward Minimizing the Impacts

Jagdish Chandra Kuniyal[1+*], Priyanka Maiti[2+*], Sandeep Kumar[3], Anand Kumar[4], Nisha Bisht[5], K. Chandra Sekar[6], Satish Chandra Arya[7], SumitRai[8], Mahesha Nand[9]

Supplementary Table S1 Temperature: Climate profile of the Dayara Bugyal area

Year	Jan	Feb	March	April	May	June	July	August	Sep	Oct	Nov	Dec	Tem mean
1901	-2.0	-1.2	4.7	8.2	11.8	15.2	15.2	14.3	12.1	9.6	4.6	1.8	8.9
1902	0.7	1.5	6.2	9.2	12.5	13.7	14.5	14.5	11.8	8	4	0.8	8.6
1903	-0.7	-0.4	2.8	7.4	11.5	15.2	15.8	13.9	12.7	8.3	3.5	0.7	7.8
1904	-1.3	0.5	3.8	9.7	11.8	14.4	14	13.7	11.6	8.1	3.5	1.4	8.9
1905	-2.5	-3.5	1.0	6.4	12.9	15	14.9	14.7	12.3	8.6	4.9	1.1	7.5
1906	-2.2	-2	2.4	8.3	13.6	13.7	14.9	14.1	11.6	8.7	5.2	2.4	8.5
1907	1.5	-1.5	1.5	6.9	10.5	13.6	15.6	13.3	12.5	8.9	4.8	0.5	7.9
1908	-0.2	0.5	4.3	9.8	12.0	15	13.8	12.7	11.3	7.7	3.5	0.6	8.7
1909	-1.5	-1.5	4.6	7.5	11.5	13	13.3	13.6	10.9	8.2	5.3	0.8	7.8
1910	-0.9	0.1	4.1	7.7	12.1	13.4	13.7	13.4	12.1	7.5	3.1	0.5	7.6
1911	0.1	0.1	2	8.2	12.9	14	16	14.7	11.8	8.6	1.8	0.7	8.4
1912	0.1	1.0	3.6	8.2	11.9	14.8	14.8	13.4	10.9	8.1	2.9	0.9	8.15
1913	0.1	-0.4	1.9	9.3	10.7	12.4	13.9	14.1	12	8.5	3.3	0.7	8.9
1914	1.3	-0.6	3.1	8.2	11.9	13.9	13.9	13.7	12.1	6.7	4.3	0.5	7.4
1915	-1.0	-1.8	4.1	8.5	14	14.2	15.2	14.3	13.1	9.7	4.4	1	9.1
1916	0.3	-0.5	6.8	9.8	11.5	13.4	14.1	13.4	11.7	8	2.6	0.3	8.9
1917	-0.3	0.6	3.6	5.6	8.7	13	13.7	13.1	10.9	6.9	2.5	0.9	6.2
1918	-1.8	0.5	3.7	6.4	13.6	13.4	15.8	14.6	11.8	8.3	3.7	1.0	7.35
1919	-0.9	-1.7	4.2	7.5	11.3	15.2	14.2	13.5	11.3	7.8	3.3	0.3	7.7
1920	-0.6	-1.2	3.5	7.8	8.7	12.8	14.3	13.8	12.7	9.1	4.9	1.7	8.25
1921	-0.2	-0.6	7	10.9	14.2	14.1	15.3	13.3	11.4	7	3.8	2.4	8.95
1922	-1.0	1.4	5.5	9.2	12.4	13.8	14.8	13.9	11.5	7.3	3.7	0.9	8.25
1923	-0.6	-0.6	4.8	9.7	11.4	15.6	14.1	13.2	11.7	7.2	3.4	1.1	8.4
1924	-1.5	-0.1	5.6	9.2	9.1	15.5	14.9	13.9	10.9	8.1	3.4	1.5	8.6
1925	-2.1	-1.3	5	10.5	11.6	12.4	13.3	14	12.1	8.5	2.8	0.9	9.5
1926	-0.3	1.7	2.9	5.8	9.7	15.1	15.1	13.7	11.3	7.6	1.9	0.7	6.7

1927	-1.8	-1.6	3.2	7.9	10.8	13.8	14.3	13.4	11.8	8.4	3.7	2.1	8.1
1928	-0.9	0.6	4.5	8.6	13.3	14.1	14.7	14.6	12.1	9.2	4.4	0.7	8.9
1929	-1.0	-1.5	6.3	9.6	12.9	14.2	13.9	13.3	12.5	7.8	4.2	-0.1	8.7
1930	-1.9	-1.0	5.1	8.0	11.9	13.5	13.3	13.9	12.2	8.3	3.5	1.2	8.1
1931	0.7	-1.8	3.8	10	12	14.6	14	14.5	11.8	8.2	3.7	1.7	9.1
1932	1.0	-0.4	5.2	9.2	11.1	15.5	15.1	13.5	12.4	8.3	4	0.8	8.7
1933	-2.6	0.6	4.3	6.6	9.9	13.8	14.2	12.7	10.9	7.7	3.9	2	7.1
1934	-2.0	2.0	3.6	8.8	11.3	13.7	14.5	13.4	12.6	7.4	3.6	1.7	8.1
1935	-2.4	0.3	4.5	5.5	12.4	14.8	14.2	13.2	11.7	7.6	4.1	0.8	6.5
1936	-1.9	0.3	3.5	8	13.9	12.3	13.5	13.5	11.8	8.0	3.7	0.8	8.9
1937	-1.3	0.1	3.5	7.1	11.9	14.6	14	14.4	12.1	7.0	3.2	-0.2	7.7
1938	-2	-2.1	4.8	9.6	13.7	12.6	14.7	14.4	12.5	8.6	2.6	1.2	9.1
1939	-0.1	-0.1	1.9	7	13.2	13	14.4	14.5	12.5	8.8	3.8	1.7	7.9
1940	-0.3	-0.1	2.8	7.5	12.6	14.4	15.1	13.3	11.5	8.3	4.8	0.9	7.9
1941	-1.2	1.0	6.5	11	13.3	13.4	15.3	14.7	12	10.5	4.4	2.4	10.7
1942	-1.6	0.7	5.9	10	12.6	14.8	13.7	13.1	11.1	8	5.2	0.6	9.9
1943	-0.8	0.3	5.1	7.4	11.9	13.8	14.2	13.5	12.7	8.2	4.9	2.3	7.8
1944	-1.4	-1.1	3.4	7.8	13.5	13.7	14.5	14.1	11.5	7.7	4.5	2.2	7.7
1945	-2.5	-0.8	4.8	7.1	11.6	14.3	14.4	14.2	12	7.8	4.1	1.0	7.4
1946	0.6	2.2	4	10.6	12.4	14.1	14.6	14.4	12.6	9.0	3.8	0.7	9.8
1947	-2.1	0.3	5.6	10.3	13.2	15.6	15	14.4	11.9	7.5	5.1	1.4	8.9
1948	-0.8	-0.7	3.2	10.1	13	14.6	14.2	13.3	12.2	8.9	3	1.1	9.5
1949	1	-0.5	4.6	10.1	13.5	14.1	14.1	14	13	8.1	2.7	0.2	9.1
1950	0.2	-1.7	2.8	7.3	12.5	14.7	14	13.5	10.7	7.5	3.3	-0.4	7.4
1951	-1.4	-0.6	4.7	6.3	11.5	13.6	14.9	14	12.4	10.6	5.3	2	8.4
1952	0.2	1.9	3.8	10	12.5	13.4	14.1	13.3	12.7	8.7	4.1	1.4	9.3
1953	-1.6	2	7.3	9.6	13.2	14.9	14.6	13.6	11.9	8.5	4	3.4	9.0
1954	-1.9	0.7	4.8	9.5	13.5	14.7	14.5	14.5	12.2	6.3	4	0.9	7.9
1955	-1.2	0.8	6.1	6.7	10.2	14.9	14.4	13.4	11.7	6.9	3.8	1.5	6.8
1956	-0.9	0.4	4.4	9.4	14.1	13.8	13.2	13.1	12.6	7.1	3.2	1.4	8.2
1957	-1.6	-1.2	3.1	6.9	10.1	13.2	14.7	13.6	11.3	7.8	4.4	1.5	7.3
1958	0.9	0.5	5.3	10.6	11.8	14.5	14.4	13.7	11.7	8	4.4	2.3	9.3
1959	-1	-1	5.1	9.1	11.5	14.8	13.8	13.9	12.2	9.3	4	1.9	9.2
1960	-1.4	2.8	3.4	7.5	12.4	14.8	14.1	14	12.5	7.9	3.4	1.7	7.7

1961	-0.3	-1.4	5.1	8.5	11.9	14.4	14.4	13.8	12.4	8.4	3.1	-0.3	8.4
1962	-1.4	1	4.2	9.4	11.7	14.5	15.1	14.2	11.6	7.9	4.4	0.8	8.6
1963	-1.1	1.7	3.9	8.1	10.8	14.4	15.5	13.6	11.6	9.3	5.4	1.5	8.7
1964	-1.7	0	5.9	9.5	10.2	14	13.3	14.2	11.9	8.6	3.8	1.7	9.0
1965	0.8	0	3.6	6.8	10.6	14.9	14.1	13.3	11.9	8.9	5.6	1.2	7.8
1966	-0.2	2.4	4.5	8.5	11.5	13.6	14.6	13.8	11.2	8	3.8	0.8	8.2
1967	-1.4	2.1	3.2	7.9	10.9	14.6	14.6	13.3	11.7	7.4	3.6	1.3	7.6
1968	-2.1	-2	4.1	8.3	10.9	14.4	14	13.4	12.9	7.5	4	1.6	7.9
1969	-0.7	0.7	6.7	8.3	10.6	14.4	14.3	14.1	11.8	9	5.6	2.1	8.6
1970	-0.3	-0.2	4	10	12.7	13.2	14.9	13.9	12.7	9.2	4	2.1	9.6
1971	-1.3	0.5	4.8	9.8	11	13.3	13.7	13.2	11.3	8.4	4.6	2.3	9.1
1972	0.7	-1.6	5.3	7.5	12.5	14.5	14.7	13.6	11.1	7.5	4.5	2	7.55
1973	-0.7	1.3	4	10.2	12.4	14.4	14.4	13.2	12.5	7.9	3.9	1.0	9.0
1974	-1	-1.2	5.9	10.2	11.8	13.1	13.8	13.8	11.9	8.5	3.5	0	9.35
1975	-2	-1.1	3.3	8.4	12	13	12.9	13.2	11.3	8.9	2.9	1.5	8.6
1976	0	0.3	3.7	7.9	11.3	12.4	14.3	12.4	11.1	8.3	5.2	1.6	8.1
1977	-0.5	0.2	6	8.6	9.6	12.9	13.5	13.6	11.4	8.7	5.9	2	8.6
1978	-1.5	-0.4	2.1	8.5	14.1	14.4	13.4	13.3	11.5	8.4	3.5	1.4	8.4
1979	-1	-1.3	2.6	9.7	10.3	14	14.3	14.2	11.1	9.1	5.7	2.1	9.4
1980	-0.5	1.4	3.6	10.3	13.5	13.8	13.8	13.6	11.8	8.4	4.5	1.6	9.3
1981	-0.5	1.3	3.6	9	12.3	14.1	13.7	13.9	12.1	7.7	3.1	0.6	8.3
1982	0.1	-1	1.9	7.5	9.2	13.3	14.8	13.6	11.5	8.3	4.3	1.9	7.9
1983	-1	-1.2	2.6	5.8	10.1	12.4	13.6	13.8	12.3	7.5	3.8	1.5	6.6
1984	-1.2	-1.2	6.3	8.8	13.7	15	13.3	13.8	10.8	7.8	3.7	1.9	8.3
1985	-1	1.3	6.6	9.2	12.2	13.8	13.5	13.9	12	7.2	4.4	2.2	8.2
1986	-1.3	0.1	3.6	8	9.6	13.3	13.2	13.6	11.6	7.8	4.9	-0.2	7.9
1987	-0.5	1.4	5.1	9.1	8.6	13.6	15	14.2	12.8	8.8	4.9	1.6	8.7
1988	1	1.8	4	9.9	13.2	13.5	14	13.3	12.1	8.0	4.2	1.9	8.9
1989	-2.7	-0.3	4.1	7.3	11.4	12.9	13.8	12.9	11.7	8.0	3.8	1	7.6
1990	2	0.3	2.3	7.8	12.2	14.4	13.7	13.5	12.1	7.3	4.6	1.5	7.5
1991	-2.3	0.5	4.4	7.2	11.5	13.4	15.4	13.4	12	7.4	4	1.8	7.3
1992	0.1	-0.8	4.1	8.1	10.4	13.5	13.5	13.3	11.3	8	4.3	2.3	8.05
1993	-1.1	2.4	2.3	8.1	12.3	14.1	13.6	14.1	11.6	8.2	5.2	2.2	8.15
1994	0.4	0.3	6.2	7.4	12.3	14.6	14.2	13.6	11.5	7.6	4.9	2.2	7.5

1995	-1.9	0.3	3.5	7.5	12.5	15.1	14.7	13	11.9	8.9	4.6	2	8.2
1996	-0.2	1.6	5.9	9.1	11.2	13.6	14.3	12.9	12.2	7.9	4.1	1.3	8.5
1997	-1.2	0	4.6	7.7	9.7	12.6	14.7	13.2	12	6.3	3.2	0.4	7
1998	-1.3	0.8	3.2	9	12.6	14.2	14.6	14	12.2	8.5	4.1	1.4	8.75
1999	-0.7	2.1	5.7	10.1	12.2	13.4	14.3	13.9	12.9	9.3	6.1	2.6	9.7
2000	0.1	-0.9	3.9	10.5	13.5	13.5	13.9	14	12.2	9.8	5.8	2.8	10.15
2001	-0.5	1.9	5.1	8.9	12.8	13.1	14.3	14.6	12.3	9.7	5.5	2.7	9.3
2002	-0.3	0.8	5.5	10.2	13.3	14.2	15.2	14.2	10.7	8.9	5.5	3	9.5
2003	-0.2	0.7	4.2	9.8	10.9	14.8	13.9	13.8	12.1	8.8	4.4	1.9	9.3
2004	-0.1	1.6	7.8	11	12.6	12.7	14.8	13.7	12.6	7.5	5	3.1	9.4
2005	-0.9	0.3	5.6	8.5	10.6	14.8	13.8	14.6	12.6	8.6	4.8	1.1	8.55
2006	0.8	4.1	4.8	8.7	13.5	13.2	14.6	13.8	11.9	9.4	5.5	2.2	9.0
2007	0.1	1.2	4.3	11.3	12.2	14	14.8	14.5	12.7	8.7	5.3	1.7	10
2008	-0.4	-0.2	7.1	8.5	11.4	13.9	14.2	13.3	11.4	9.3	5.5	3.3	8.9
2009	1.2	2.5	6	9.7	12.7	14.1	14.8	14.8	11.7	8.9	5	2.6	9.3
2010	-0.8	1.6	7.	12.1	13	13.8	14.7	14	12	9.5	6.1	1.6	10.7
2011	-1.5	1.3	5.2	8.5	13.3	13.5	14.4	13.8	12.2	9.2	6.4	2.6	8.85
2012	-1.4	0.4	5.2	8.9	12.5	15.5	15.6	13.8	12.3	8	4.7	2.2	8.4
2013	-1.6	-0.3	5.7	9.1	13.1	13.9	14.7	13.7	12.5	9.7	4.5	2.4	9.4
2014	-0.5	-0.2	3.9	8.3	11.4	15	15	14.5	12	9	5.3	1.4	8.65
2015	-0.5	2.1	3.9	9.1	12.8	13.1	13.7	13.9	12.5	9.5	5.8	2.5	9.3
2016	0.8	2.8	6.5	10.7	13.4	15.1	14.6	13.9	13.1	10.1	6	4.2	10.4
2017	0.2	2.9	6	10.9	12.8	13.1	14.6	14.4	12.9	10.3	4.8	3.2	10.6
2018	1.4	3.1	7.4	10.5	12.8	14.4	14.5	14.4	12.1	8.3	4.9	1.4	9.4
2019	-0.8	0.1	4	10.5	11.7	14.2	14.5	14.1	13.3	8.8	5.3	0.3	9.6

Source: CRU TS 4.04 (land), 0.5°

Supplementary Table S2 Precipitation: Climate profile of the Dayara Bugyal area

Year	Jan	Feb	March	April	May	June	July	August	Sep	Oct	Nov	Dec	Precipitation
1901	98.5	65	52.2	7.9	49.9	36.9	232.8	269.7	65.4	3.2	0.7	1.1	51.05
1902	3.0	11.1	23.4	52.4	41.1	80.4	237.7	149	115.7	18.8	0.7	19.9	32.2
1903	44.3	11.2	53	13.9	37.2	30.3	154.6	206.9	100.1	39	0.8	22.8	38.1
1904	31.1	7.7	88.8	8.2	44.6	83.2	234.6	239.7	120.4	13.6	27.4	22.1	37.8
1905	99.8	82.8	46.3	17.6	19.2	28.4	182.2	137.6	90.0	3.2	0.8	15.6	37.3
1906	27.7	128.5	82.7	10.3	10.6	155.7	135.2	156.5	186.9	4.9	0.7	1.1	55.2

1907	47.3	84.0	109.1	105.5	22.4	36.6	157.4	213.1	17.2	3.4	0.7	7.7	41.9
1908	59.9	57	12.1	42.2	28.6	39.1	224.6	288.4	48	2.1	5.4	51.2	45.1
1909	63.1	40.1	6.4	94.6	22.6	214	352	180.3	93.7	11.4	0.7	17.5	51.6
1910	36.6	34.7	11.7	13.1	25.8	147.4	300.7	243.4	176.6	47.2	1.9	2.4	35.6
1911	99.8	10.1	128.9	24.4	6.2	106.1	67	170.1	175.3	33.1	61.4	7.9	64.2
1912	66.8	32.7	28.3	51.4	39.2	52.7	211.1	225.4	185.2	2.6	28.4	36.1	45.3
1913	10.3	99.3	71.1	14.2	86	189.6	142.9	138.4	26.9	4.9	9.4	11.6	49
1914	4.6	65.1	52.9	69.5	48.5	109.5	291	188.9	297.4	51.7	31.8	12.9	59
1915	62.5	88.5	85.5	42.8	22.1	71.3	142.1	289.6	120.6	12.1	0.7	1.6	66.95
1916	5	48.8	8.3	19.7	36.2	182.1	256.8	176	152.1	47.5	0.7	12.3	41.85
1917	19.2	39.8	46.2	111.9	90.8	161.4	271.1	193.9	287.1	89.4	0.7	5.5	90.15
1918	16	4.2	62.9	82.8	15.6	115.2	146.6	172.7	27	6.2	21.4	18.4	24.2
1919	90.8	26.4	35.7	53.9	43.9	80.6	307.1	174.1	74.9	10.6	7.8	3.1	48.9
1920	26.2	37.8	56.9	7	65	153.1	301.2	104.7	73.2	4.6	0.7	14.3	47.35
1921	68.9	13.4	6.9	11	5.6	41.5	196.4	267	145.4	40.9	0.7	50.5	41.2
1922	68.2	28.6	10.2	13.6	7.5	75.7	267.6	308.4	185.9	14.2	4.1	38.1	33.35
1923	40.9	121.4	10.9	9.6	34.9	51.1	244.4	221.7	113.8	51.8	21.1	32.9	46
1924	54.8	60.4	18.6	10.2	34.6	32.8	239	197.4	294.6	56.5	4.3	1.1	44.7
1925	32.1	7.7	11	21.2	62.1	186.6	296	215.4	45.7	16.3	35.2	5.9	33.6
1926	26.1	17.2	117.8	55.7	57.8	19.6	284.7	258.3	73.5	8.1	15.3	20.3	40.9
1927	5.1	79.7	32.9	25.6	56.5	34.6	197.2	239.8	83.1	83.2	7.1	41.7	49.1
1928	72.3	104.6	27.4	51.5	11.8	59.8	189	137.8	47.2	10.6	25.9	55.7	53.6
1929	59.2	8.6	14.1	38.2	14.6	82.6	200.1	205.1	43.7	36.1	0.7	24.8	37.15
1930	50.7	41.4	16.7	28.8	22.8	113.8	253.4	170.1	73.2	25.3	11.7	1.4	35.1
1931	9.3	63.4	35.2	10.2	37.9	24.7	179.8	162.4	197.8	76.3	2	30.9	36.55
1932	9.1	16.3	36.2	16.4	23.6	59.9	147	149.8	215	12.4	1.9	2.2	20
1933	24.3	60	31.7	38.4	73.6	136	214.8	246.1	213.2	77.3	2.2	38.5	66.8
1934	59.5	6.7	67.8	8	22.9	109	253.8	251.2	42.5	1.6	0.7	25.5	34
1935	81.9	51.8	22.6	55.7	5.8	36.1	203.3	188.8	117.4	8.1	20.8	64.6	53.75
1936	6.1	56.3	37.1	37.4	44.4	200.4	336.5	202.9	133.6	12.9	5.1	33.3	40.9
1937	14.7	122.4	11.1	66.5	34.4	103.9	233.8	145.1	156.6	17	2	1.8	50.45
1938	102.8	49.2	15.6	17.9	10.7	142.2	206	182.9	37.6	15.3	4.8	1.1	27.75
1939	19.2	59.1	50.2	28.1	8.9	135.6	197.9	106	84.9	16	0.7	14.5	39.15
1940	43.2	87.5	40.2	34.6	21.1	83.1	228.9	197.3	56.5	6.1	0.8	21.1	41.7
1941	57.9	26.3	18.2	3.0	50.8	105	122.6	191.2	79.5	21.5	2.2	39.4	45.1
1942	55.2	79.3	15.4	41.5	29.2	91.3	326.6	235.4	145	4.5	0.7	2.2	48.35

1943	65.8	7.7	23.8	64.5	19.4	85.8	170	274.8	98	1.6	0.7	13.1	44.15
1944	64	60.9	82.6	84.7	10.4	103.4	168	138.4	100.1	21.1	2.8	2.2	73.3
1945	70.7	3.8	21.2	36.8	18.4	48.2	262.3	196.8	218.2	55.7	0.7	25.3	42.5
1946	6.2	35.1	14.6	44.9	58.2	107.8	259.4	139.9	43.6	68	16.9	23.4	44.2
1947	31.3	39.3	55.2	6	22	35	152.9	169.5	285.6	23.8	1.8	13.4	33.15
1948	27.2	70.1	91.3	9.9	13	27.8	212	299.6	96.2	21	0.9	7.1	27.5
1949	15.4	67.5	22.8	35.6	30.2	42.6	353.9	151.6	126.6	19.9	1.2	4.6	32.9
1950	74.8	29.8	66.6	6.9	33.4	117.7	319.5	206.2	182.7	3.4	1.2	1.4	50
1951	48.9	22.7	95.4	42.2	33.6	41.3	156.6	195	113.4	11.5	44.9	7.5	43.55
1952	38.2	36.3	76.7	23.8	46.8	131.5	143.7	252.9	21.4	2.1	9.4	3.3	37.25
1953	86.2	15	6.5	32.3	13.8	111.8	339.6	210.1	53.3	2.6	0.9	2.9	23.65
1954	65.8	125.5	33	3.7	9.5	49.4	265.9	217	156.7	43	0.7	2.4	46.2
1955	72.1	10.7	41	44.9	30.7	89.6	230.2	231.9	164.6	126	0.7	17.3	58.5
1956	35.5	11.1	63.3	13.5	51.6	114	191.5	204.1	101.7	87.2	2.7	46.5	57.45
1957	87.8	4.2	60.7	30.9	27.3	66	253.1	159.1	137.5	24.7	17.6	27.9	45.8
1958	44.5	15.1	32.2	17.5	12.6	52.4	270.4	228.6	193.6	62.8	2.6	1.4	38.35
1959	61.7	38.7	38.2	20.8	43.4	83.1	255	175.2	141.9	42.9	16.4	13	43.1
1960	52.9	3.1	53.6	18	19.8	70.9	249.8	198.6	85.5	55.7	0.7	28.5	53.25
1961	77.4	78.5	16.3	18.5	20.7	95	215.2	211.7	91.3	56.2	4.6	20.7	66.8
1962	82.2	39.7	46	25.5	23.8	83.7	199.5	155.5	244.4	2.1	13.1	21.4	42.85
1963	11.5	44.7	118.2	33.8	39.6	76	161.6	243.7	160.5	9.7	22.2	13.6	42.1
1964	21.7	16.7	24.5	18.8	52.5	53.9	309.1	204.3	144.6	2	5.2	1.6	23.1
1965	22.5	68	49.5	49	35	33.6	197.8	163.5	58.8	36.6	5.6	8.2	42.8
1966	6.9	48.3	41.2	24.8	44.1	142.2	156.7	248	80.5	36.4	19.3	38.7	42.6
1967	14.2	30.9	82.5	16.4	10.1	74.1	213.2	223.6	87.8	19.4	5.8	15.9	25.15
1968	46.2	37.9	31.3	22.2	7.1	97.8	244.2	180.2	62.8	25.3	3.2	1.7	34.6
1969	22.9	25.4	39.9	62.8	42.2	53.6	200.8	223.7	218.5	7.4	1.5	1.4	41.05
1970	54.3	37.7	37.9	3.2	53.9	130.8	152.6	209.4	131.9	20	0.7	1.8	45.9
1971	30	43.3	22.8	54.2	109.2	194.5	209.8	251	134.8	51.4	12.3	8.9	52.8
1972	20	46.4	21	37.7	12.9	58.3	201.8	168.9	200.6	42.7	20.6	12.9	40.2
1973	48.9	32.2	35.3	6.6	40.4	194.7	169.7	222.9	88.2	64.6	2.7	18.4	44.65
1974	17.5	22.8	20.6	10	42.4	87.7	174.4	146.1	66	16.4	0.7	1.1	21.7
1975	62.8	38	50.3	4.0	17.5	139.2	173.9	229.9	178.9	44	0.7	3.2	47.15
1976	19.1	64.7	23.5	26.1	43.8	183.5	199.4	245.5	62.4	11.7	0.7	26.4	35.1
1977	53.1	7.7	6.5	89.8	62.5	160	262.9	148.9	125.2	17.3	1.6	9.3	57.8
1978	10.2	47.4	95.2	36	14.8	134.8	218.5	244.5	189.8	6.1	29.8	18.1	41.7

1979	42.6	80.9	42.8	40.4	50.2	72.4	190.6	107.6	70.8	8.1	16.2	23.4	46.5
1980	10.9	35.9	55.6	13.1	35.6	107	341.2	173.1	99.2	18.9	10.8	6.2	35.75
1981	48.3	22.3	70.9	27.4	57	84.6	211.4	141.3	61	2.1	49.1	18.5	53.05
1982	44.3	32.4	104.4	97.9	70.2	71.5	152.7	184.1	41.4	16.2	6.9	10.3	57.25
1983	59.5	26.3	50.6	95.7	68.8	122.1	185	176.8	138.9	56.9	4.3	16.6	64.15
1984	14.9	82.6	12.6	38.7	29	114.8	182.2	179.5	113	3.5	2.4	36.4	37.55
1985	37.9	4.8	13.8	62.1	31.4	95.7	341.7	201.9	122.1	92.4	1.6	42.2	52.15
1986	11.1	36.2	51	36	70.4	73.8	205.2	151.6	124.6	29.9	11.9	21.2	43.6
1987	45.3	37	33.4	37.2	104	39.4	130.6	158.4	47.9	30.3	0.7	33.6	38.3
1988	12.6	28.7	68.5	30.2	31.4	111.3	274.5	243.4	175.5	2.4	1.2	12.5	30.8
1989	100.7	19.3	41.3	6.2	23.7	56	256.6	179.6	102.2	9.6	25.5	39.3	40.3
1990	6.3	99.2	73.9	16.3	102.9	76	246.2	223.3	114.1	7.2	12.9	18.6	74.95
1991	6.3	46.9	26	34.7	47.1	84.4	76.5	203.3	84.3	2.7	16.6	1.8	40.8
1992	48.5	62.2	47.9	10.6	28.6	66.8	182.8	138.6	65.5	12.2	47.9	1.6	48.2
1993	41.6	37.7	41.7	14.8	28.3	173.4	203.9	85.4	164.3	2.2	1.1	3.5	39.65
1994	47.5	38.2	10.4	33	45.3	51	292.7	224.4	31.1	2.9	1.9	2.9	35.6
1995	83.5	94.2	37.1	20.6	7.1	79.8	165.6	258.6	119.1	4.4	33.3	1.6	58.45
1996	32.3	45	48.8	54.5	14.1	141.5	118	176.6	123.2	33.2	0.9	63.7	51.65
1997	17.7	9.4	36.6	89.7	54.4	135.9	118.1	144.6	52.7	50.8	43.2	1.6	51.75
1998	4.7	43.1	67.8	37.8	25.8	87.9	147.1	193.1	97.9	57.6	13.8	6.1	50.3
1999	49.5	35.4	7	23.8	25.6	103.2	176.6	220.3	116.8	22.4	0.9	14.7	30.5
2000	25.1	105.5	27.7	30.5	122.1	207.2	385.7	119.3	46.2	2.9	19.4	3.8	38.35
2001	39.7	33	15.7	58.1	112.9	159.1	192.3	72.9	32.5	5.1	2.8	8.4	36.35
2002	28	83.1	39.6	79.7	9.8	151.6	55.9	247.6	196.8	6.7	6.8	20.1	47.7
2003	47.4	105.5	22.2	58.1	13.6	82.8	218.7	126.2	67.3	14.9	13.2	18	52.7
2004	14	7.3	4.2	9.1	32.2	209.6	54.8	105.8	21.3	57.3	1.7	5.7	17.65
2005	18.6	62.2	37	20.6	15.4	95	161	76.8	203.4	11.4	1.3	7.8	28.8
2006	5.5	4.3	38.8	8.5	70	74	256.9	171.4	82.3	44	9.1	3.0	41.4
2007	4.1	65.9	102.4	77.3	114.9	71.8	80.5	128.7	70.4	4	3.6	5.2	71.1
2008	37.5	11.8	15.2	48.8	22.1	169.6	120.8	187.2	120.9	5.9	2.8	2.8	29.8
2009	8.5	31.6	12	20.1	91.3	17.0	85.7	76.9	196.4	44.5	22.9	12.1	27.25
2010	52.2	60.6	4.3	9.6	13.6	23.8	189.1	237.1	134.6	10.3	17	3.6	20.4
2011	12.2	75.4	30.4	28.8	60.3	229.3	188.6	205.6	103.1	3.5	4.2	11.2	45.35
2012	37.1	6.7	22.1	51.1	6.3	15.3	107.9	264.2	153.7	5.4	4.5	7.4	18.75
2013	65	141.4	18.6	37	11.3	146.0	203.6	201.2	93.5	27.9	4.4	10.5	51
2014	35.3	94.2	45.3	26.2	42	69.3	181.8	148.2	65.2	45.4	1.5	2.5	45.35

2015	48.4	45.4	135.1	65.6	12.5	228.4	303.8	240.8	38.7	7.1	3.9	1.6	46.9
2016	5.6	14.8	42.1	11.1	65.1	76.0	307.6	136.8	47.8	7.7	1.3	6.8	28.45
2017	28.9	7	18.6	128.1	81.8	190.8	356.5	162.2	118	2.2	10	8.1	55.35
2018	9.6	7.2	43	24.4	20.7	109.3	246.1	210.1	168.4	5.1	43.1	49.2	43.05
2019	66.5	85.3	23.7	41.8	25.8	49.6	312.1	210.1	87.8	20.4	31.1		49.6

Source: CRU TS 4.04 (land), 0.5°

Supplementary Table S3 Vegetation analysis of the grazing land in the Papad Gad and Swari Gad area of the Dayara Bugyal

Total species in the sampled grazing land	Density (individual /m^2)
Aconitum heterophyllum Wall. *ex* Royle	0.27
Aconogonum tortuosum (D. Don) Hara	0.4
Allium humile Kunth	0.25
Allium stracheyi Baker	0.24
Aletris pauciflora (Klotzcsh) Hand.-Mazz	0.2
Anaphalis cuneifolia Hook. f.	0.73
Anaphalis royleana DC.	0.24
Anemone obtusiloba D.Don	0.23
Anemone rivularis Buch.-Ham. *ex* DC.	0.64
Angelica glauca Edgew.	0.15
Arctium lappa L.	0.3
Arnebia benthamii (Wall. ex G. Don) I.M. Johnst.	0.06
Artemisia vestita Wall. *ex* Besser	0.43
Astragalus chlorostachys Lindl.	0.23
Bergenia stracheyi (Hook. f. & Thomson) Engl.	0.17
Bistorta amplexicaulis (D. Don) Greene	0.24
Bistrota affinis (D. Don) Greene	0.2
Bistorta vivipara (L.) S.F. Gray	9.8
Bupleurum longicaule Wall. *ex* DC.	0.2
Caltha palustris L.	0.27
Carex nubigena D. Don	4.42
Carex setigera D. Don	0.82
Cyananthus integer Wall. *ex* Benth.	0.23
Cyananthus lobatus Wall. *ex* Benth.	0.57
Dactylorhiza hatagirea (D.Don) Soo	0.23
Danthonia cachemyriana Jaub. &Spach	0.33
Dolomiaea macrocephala DC.	0.23
Epilobium latifolium L.	0.24
Eritrichium canum (Benth.) Kitam.	0.62
Euphorbia stracheyi Boiss.	0.2
Galium rotundifolium L.	0.23
Gentiana argentea (D.Don) Griseb.	0.23

Geranium wallichianum D.Don *ex* Sweet	0.3
Geum elatum Wall. *ex* G. Don	0.2
Hackelia uncinata (Royle *ex* Benth.) C.E.C. Fisch.	0.57
Impatiens scabrida DC.	0.31
Impatiens sulcata Wall.	0.28
Iris kemaonensis Wall. *ex* Royle	0.6
Kobresia nepalensis (Nees) Kük.	0.43
Morina longifolia Wall. *ex* DC.	0.37
Origanum vulgare L.	1.22
Oxygraphis polypetala (D.Don) Hook.f. & Thompson	0.02
Parnassia nubicola Wall. *ex* Royle	0.27
Picrorhiza kurrooa Royle *ex* Benth.	0.08
Poa alpina L.	0.23
Polygonatum verticillatum (L.) All.	0.25
Polygonum polystachyum Wall. *ex* Meissn.	0.4
Potentilla argyrophylla Wall. *ex* Lehm.	0.5
Potentilla atrosanguinea Lodd. *ex* Lehm.	0.3
Potentilla fulgens Wall. *ex* Hook.	0.13
Primula denticulata Sm.	0.4
Prunella vulgaris L.	4.43
Ranunculus hyperboreus Rottb.	0.4
Ranunculus hirtellus Royle	0.5
Rhodiola trifida (Hook. f. & Thomson) Jacobsen	0.33
Rumex nepalensis Spreng.	1.19
Salix lindleyana Wall. *ex* Andersson	0.01
Saxifraga parnassifolia D.Don	0.47
Selinum wallichianum (DC.) Raizada & H.O. Saxena	0.4
Sibbaldia parviflora Willd.	0.57
Swertia ciliata (D. Don *ex* G. Don) B.L. Burtt	0.47
Tanacetum longifolium Wall. *ex* DC.	0.32
Taraxacum officinale W.W. Weber *ex* F.H. Wigg.	9.79
Thalictrum foliolosum DC.	0.3
Thermopsis barbata Benth.	0.48
Thymus linearis Benth.	0.23
Trachydium roylei Lindl.	19.33
Trifolium repens L.	0.53
Valeriana hardwickii Wall.	0.04
Viola biflora L.	4.24

Supplementary Table S4 Migratory livestock data of Tankunr range

Year	Buffalo	Cow/Ox	Horse	Sheep/Goat	Total Animal unit
2003-04	458	24	99	32	606.1
2004-05	495	32	88	32	638.2
2005-06	462	35	102	61	631.3
2006-07	489	27	101	84	654.5
2007-08	409	91	59	57	582.7
2008-09	441	77	80	90	633.1
2009-10	478	105	96	106	720.6
2010-11	397	87	83	80	600.3
2011-12	411	89	62	65	588.1
2012-13	383	104	72	81	590.5
2013-14	419	102	88	84	644.1
2014-15	379	153	78	74	641.0
2015-16	423	174	108	97	746.7
2016-17	433	162	99	64	726.5
2017-18	415	143	82	28	661.2
2018-19	323	92	61	20	491.5
2019-20	338	95	76	18	526.8

Source: Forest department entry registrar, Uttarkashi, 2020

Supplementary Table S5 Time series analysis of the Dayara Bygyal tourists' influx

Year	Number of Tourists	Forecast (Number of Tourists)	Lower Confidence Bound(Number of Tourists)	Upper Confidence Bound (Number of Tourists)
2015	7000			
2016	15000			
2017	13000			
2018	17500			
2019	20000	20000.00	20000.00	20000.00
2020		23775.70	20186.07	27365.34
2021		21624.35	18005.89	25242.83

2022		28463.33	24815.36	32111.31
2023		26311.98	22634.73	29989.23
2024		33150.95	29443.76	36858.15
2025		30999.60	27262.71	34736.51

Source: Tourism department, Uttarkashi, 2020

Supplementary Table S6 Amount of soil checked from erosion by the chek-dams

Geo-Check dam						Bulk density or Mg/m^3			Mean BD	Mass of the debris in the check dam (Mg) Tonne
Swarigad Dayara Bugyal										
Brook (Nala) Number	Geo-Dam Number	Measurements			Total					
		Length	Breath	Depth						
First	17	2.50	1.60	0.213	0.85	1.45	0.96	1.68	1.37	1.17
		2.50	1.60	0.29	1.16	1.87	1.78	1.09	1.59	1.84
		1.40	1.60	0.68	1.52	1.67	1.85	1.06	1.53	2.33
		2.30	1.70	0.153	0.59	1.69	1.063	1.57	1.44	0.86
		4.00	2.40	0.13	1.30	1.95	2.03	1.94	1.98	2.59
		4.00	2.30	0.19	1.77	1.97	1.84	1.73	1.85	3.29
		2.00	2.00	0.19	0.77	1.79	1.92	1.75	1.82	1.41
		4.30	1.60	0.3	2.06	1.86	1.87	0.98	1.58	3.25
		7.30	1.80	0.16	2.10	1.96	1.02	1.10	1.36	2.87
		2.00	0.80	0.16	0.26	1.34	1.86	1.92	1.71	0.45
		1.10	1.20	0.34	0.45	1.56	2.79	1.85	2.07	0.94
		1.10	1.30	0.16	0.23	1.01	1.26	1.35	1.21	0.29
		4.30	2.00	0.17	1.46	1.88	1.35	1.45	1.57	2.29
		2.00	1.20	0.24	0.57	1.68	1.01	1.57	1.43	0.82
		2.50	1.50	0.26	0.99	1.70	1.85	1.02	1.53	1.52
		2.30	1.30	0.22	0.66	1.99	1.78	1.99	1.92	1.28
		3.30	1.40	0.22	1.04	1.98	1.25	1.47	1.57	1.64
				Total	17.85M3					
	15	2.60	3.70	0.43	4.16	1.81	2.09	1.12	1.67	6.97
		3.00	3.80	0.3	3.42	2.08	2.45	1.85	2.13	7.28

Second		3.30	3.80	0.4	5.01	1.08	1.34	2.55	1.66	8.31
		3.60	2.30	0.26	2.20	1.10	2.69	1.55	1.78	3.93
		3.20	2.10	0.26	1.78	1.77	2.85	1.19	1.94	3.46
		4.90	2.10	0.18	1.88	1.91	1.02	1.07	1.33	2.51
		10.00	2.10	0.16	3.48	1.87	1.43	1.45	1.58	5.52
		5.00	2.10	0.07	0.81	1.70	1.07	1.35	1.37	1.10
		6.50	3.00	0.2	3.90	1.62	1.29	1.14	1.35	5.27
		6.00	2.10	0.21	2.72	1.75	2.76	2.36	2.29	6.23
		11.80	2.70	0.33	11.15	1.68	2.60	1.17	1.82	19.10
		8.80	2.10	0.2	3.69	1.94	1.67	1.09	1.57	5.79
		4.30	2.10	0.15	1.35	2.75	2.92	1.97	2.54	3.45
		5.10	4.80	0.18	4.47	1.08	2.66	1.69	1.81	8.11
		7.00	2.10	0.13	1.95	1.44	2.64	1.44	1.84	3.60
									Total	169.64
Third	6	2.40	2.10	0.26	1.34	2.672	2.85	2.67	2.73	3.67
		2.70	1.20	0.13	0.43	2.90	1.41	1.92	2.08	0.90
		5.00	1.10	1.00	5.51	1.11	1.75	2.67	1.85	10.16
		3.00	2.10	0.66	4.15	2.39	1.65	2.69	2.25	9.35
		6.00	2.10	0.7	8.82	1.76	1.34	1.58	1.57	13.81
		4.50	2.10	0.8	7.56	1.60	1.32	1.95	1.63	12.29
				Total	27.80M3					

Supplementary Table S7 Socio economic survey assessment of the Dayara restoration work

Economic feasibility			
	Satisfactory	Average	Not Satisfactory
Economic efficiency	28	32	0
Cost effectiveness	36	10	14
Political feasibility			
	Satisfactory	Average	Not Satisfactory
Governance mechanism	20	30	10
Exsiting policy & legislation	21	19	20
Technical feasibility			

	Satisfactory	Average	Not Satisfactory
Technical sophistication	33	12	15
Replicability	47	13	0
Adoption lag	40	15	5
Cultural acceptability			
	Satisfactory	Average	Not Satisfactory
Traditional knowledge & practices	33	17	10
Cultural values & social norms	25	25	10
Social acceptability			
	Satisfactory	Average	Not Satisfactory
Social preference	30	10	10
Procedural equality	10	50	0
Distributional equality	8	40	2

Supplementary Table S8. Village population of Barsu and Raithal village assessed during survey

Raithal Village			Age groups of survey			
Total Household	192		20 - 35	36 - 45	46 - 60	>60
Total Population	1005	Male	14	18	12	16
Male	501	Female	12	24	16	8
Female	504					
Barsu Village						
Total Household	98		20 - 35	36 - 45	46 - 60	>60
Total Population	469	Male	8	28	12	12
Male	228	Female	24	12	12	12
Female	2**41**					

Supplementary Table S9 Index scoring details ofthe studied zones in the Daraya Bugyal

Category score			
Name of category	Reference zone	Treated zone	Untreated zone
Direct management measure (M)	2.6	1.87	1
Environmental desirability (E)	2.25	1.8	1.13

Name of zone	Eco-system index score
Reference zone	81.94
Treated zone	64.5
Untreated zone	52.03

Supplementary S10 Socio-economic survey questionnaire

G.B. Pant National Institute of Himalayan Environment,

Kosi-Katarmal, Almora, Uttarakhand

UNDP SECURE – Himalaya

(Socio-economic response of Dayara restoration)

Project Title:*Undertaking a study to define the extent and intensity of habitat degradation, to identify the drivers of degradation and prepare a study proposal for developing model mitigation plan in alpine and sub-alpine areas in SECURE Himalaya project landscapes of Uttarakhand.*

Name नाम................................ Age आयु..........................

Sex लिंग..................................Addressपता..................................

Villageगाँव..

Education शिक्षा...

Index parameter	Class	Variables	Satisfactory	Average	Not Satisfactory
Socio-economic feasibility (SE)<br>सामाजिक-आर्थिकव्यवहार्यता (एसई)	SE1: Economic feasibility (E)<br>एसई 1: आर्थिक व्यवहार्यता (E)	E1: Cost-effectiveness<br>ई1: लागत-प्रभावशीलता			
		E2: Economic efficiency<br>ई 2: आर्थिकदक्षता			
	SE2: Social acceptability (F)<br>एसई 2: सामाजिक स्वीकार्यता (F)	F1: Procedural equity<br>एफ 1: प्रक्रियात्मक इक्विटी			
		F2: Social preference			

		एफ 2: सामाजिकप्राथमिकता			
	SE3: Technical feasibility (G)<br>एसई3: तकनीकीव्यवहार्यता (G)	G1: Adoption lag<br>जी1: दत्तक ग्रहण			
		G2: Replicability of the response<br>जी2: प्रतिक्रिया की पुनरावृत्ति			
		G3: Technical sophistication<br>जी 3: तकनीकीपरिष्कार			
	SE4: Cultural acceptability (H)<br>एसई4: सांस्कृतिकस्वीकार्यता (H)	H1: Cultural values<br>एच 1: सांस्कृतिकमूल्य			
		H2: Social norms<br>एच2: सामाजिकमानदंड			
	SE5: Political feasibility (I)<br>एसई5: राजनीतिकव्यवहार्यता (I)	I1: Policy/legislation and<br>आई1: नीति / कानूनऔर			
		I2: Governance mechanism<br>आई 2: शासन तंत्र			

Satisfactory=3, Average=2, Not Satisfactory=1

Signature

हस्ताक्षर
